# LectinOracle: A Generalizable Deep Learning Model for Lectin–Glycan Binding Prediction

**DOI:** 10.1002/advs.202103807

**Published:** 2021-12-04

**Authors:** Jon Lundstrøm, Emma Korhonen, Frédérique Lisacek, Daniel Bojar

**Affiliations:** ^1^ Department of Chemistry and Molecular Biology University of Gothenburg Gothenburg 41390 Sweden; ^2^ Wallenberg Centre for Molecular and Translational Medicine University of Gothenburg Gothenburg 41390 Sweden; ^3^ Swiss Institute of Bioinformatics Geneva 1227 Switzerland; ^4^ Computer Science Department UniGe Geneva 1227 Switzerland; ^5^ Section of Biology UniGe Geneva 1205 Switzerland

**Keywords:** bioinformatics, carbohydrate, computational biology, glycobiology, machine learning

## Abstract

Ranging from bacterial cell adhesion over viral cell entry to human innate immunity, glycan‐binding proteins or lectins are abound in nature. Widely used as staining and characterization reagents in cell biology and crucial for understanding the interactions in biological systems, lectins are a focal point of study in glycobiology. Yet the sheer breadth and depth of specificity for diverse oligosaccharide motifs has made studying lectins a largely piecemeal approach, with few options to generalize. Here, LectinOracle, a model combining transformer‐based representations for proteins and graph convolutional neural networks for glycans to predict their interaction, is presented. Using a curated data set of 564,647 unique protein–glycan interactions, it is shown that LectinOracle predictions agree with literature‐annotated specificities for a wide range of lectins. Using a range of specialized glycan arrays, it is shown that LectinOracle predictions generalize to new glycans and lectins, with qualitative and quantitative agreement with experimental data. It is further demonstrated that LectinOracle can be used to improve lectin classification, accelerate lectin directed evolution, predict epidemiological outcomes in the context of influenza virus, and analyze whole lectomes in host–microbe interactions. It is envisioned that the herein presented platform will advance both the study of lectins and their role in (glyco)biology.

## Graphical abstract

Using a large, curated data set of protein–carbohydrate interactions, a new deep learning model, LectinOracle, is introduced. LectinOracle predicts protein–carbohydrate interactions from their sequences and generalizes to new proteins, carbohydrates, and contexts. Applying LectinOracle to the microbiome and virus epidemics demonstrates its utility for analyzing carbohydrate‐binding proteins in health and disease.

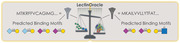

## 1 Introduction

Lectins, a class of glycan‐binding proteins (GBPs), are present across all domains of life and play a fundamental role in a diverse range of biological functions by recognizing and binding specific carbohydrate structures on cell surfaces.^[^ ^1^ ^]^ Examples of their importance in biology abound.^[^ ^2^ ^]^ Following infection, activation of the complement pathway is regulated by mannose‐binding lectin (MBL) recognizing mannose residues on the surface of pathogens. Homing of leukocytes during an adaptive immune response is coordinated through expression of selectins on the activated endothelium at the site of infection.^[^ ^3^ ^]^ Viral and bacterial pathogens in turn use lectins to adhere to and infect target cells. The host infection by influenza viruses is mediated by hemagglutinin that binds sialic acid on the surface of cells in the upper respiratory tract. In fact, recognition of sialic acid in different contexts by different hemagglutinin sequences forms the basis for influenza host range.^[^ ^4^ ^]^

Lectins are often divided into many families based on sequence similarity and, consequently, common structural folds. Ligand binding is commonly enhanced by the presence of repeated glycan‐binding domains (GBDs) and assembly into multimeric complexes, thus increasing the overall avidity for the cognate glycan.^[^ ^5^ ^]^ While GBPs are found in all domains of life, the distribution of lectin families varies across taxonomic groups, suggesting the independent emergence of glycan‐binding mechanisms during evolution.^[^ ^6^ ^]^

Glycans, the specific ligands of lectins, are composed of monosaccharides assembled into complex, branching structures and are present on the surface of all cells.^[^ ^7^ ^]^ The composition and structure of glycans varies between cell types, species, and disease state, resulting in differential preferences for lectin interactions, particularly in complex samples.^[^ ^8^ ^]^ Despite rapid methodological development in recent years, the experimental study of glycan structure and function remains limited compared to analogous investigations of DNA, RNA, and proteins, revolutionized by the emergence of affordable next‐generation sequencing and high‐sensitivity mass spectrometry. While sequential in nature, glycans are not included in the central dogma of biology and thereby do not benefit from sequencing‐based approaches. Structural and functional investigations of glycans are further complicated by: i) non‐templated synthesis regulated by subcellular localization and expression levels of glycosyltransferases, glycosidases, and nucleotide sugar substrates, and ii) nonlinear structures with multiple possible branch points and different linkages.

The intrinsic ability of lectins to selectively bind specific glycan motifs presents an opportunity to perform functional glycan investigation without the need for explicitly determining monosaccharide sequences. In experimental settings, lectins are used, e.g., for characterizing molecular interactions, investigating cell identities, and mitogenic stimulation.^[^ ^1^ ^]^ In a clinical context, the glycan‐binding properties of lectins can be exploited in the development of therapeutic strategies,^[^ ^9^, ^10^ ^]^ e.g., neutralization of bacterial toxins, or interfering with cellular adhesion, thus preventing viral infection. However, only a fraction of the known lectins has been experimentally characterized regarding glycan‐binding specificities, limiting the readily available resources for experimental studies of glycan function.

Recently, ≈1,000,000 putative lectin sequences were identified from UniProt data sets of >24,000 species, providing a database of possible lectins, e.g., for use in future biochemical analysis and therapeutics development.^[^ ^11^ ^]^ To use these lectins, their binding specificity has to be established. In the past two decades, glycan‐binding profiles of lectins have been mapped using glycan arrays. Here, hundreds of distinct glycan structures are immobilized on a glass surface to quantify the glycan‐binding ability of a specific protein sample. While providing accurate binding specificities, the individual experimental investigation of thousands of lectins using glycan arrays is currently not feasible. However, computational predictions informed by machine learning models could narrow down the experimental search space.

Machine learning algorithms provide predictions for unseen input data, based on relationships learned from labeled training data. Deep learning structures such algorithms in layers, enable the identification of salient features without having to explicitly designate them. This creates a model that can be optimized and ultimately outperformed human ability in tasks such as language processing or computer vision.^[^ ^12^, ^13^ ^]^ Recent advances in deep learning have provided neural net architectures capable of solving highly complex biological problems. Accurately predicting protein structure from amino acid sequence, seeming like an insurmountable task a few years ago, is now readily available with tools such as AlphaFold2 and RoseTTAFold.^[^ ^14^, ^15^ ^]^ Deep learning models such as Evolutionary Scale Modeling 1b (ESM‐1b)^[^ ^16^ ^]^ have been trained on the entirety of UniRef50, millions of clustered protein sequences, to learn relationships of the protein space and use this understanding for predicting structural or functional properties of these sequences. In the case of ESM‐1b, a transformer‐based model learns relevant sequence stretches from proteins via the mechanism of attention to understand protein similarity. This is performed by using single protein sequences as inputs to a trained ESM‐1b model and receiving a learned representation, a vector of numbers positioning this protein in a multidimensional learned space that can be used for predictions.

In glycobiology, deep learning has recently enabled new analyses of sequence–function relationships.^[^ ^17^, ^18^ ^]^ Based on this, we developed SweetNet,^[^ ^8^ ^]^ a graph convolutional neural network method that learns glycan representations by taking their branching structures into account. Briefly, SweetNet considers glycans as molecular graphs, with monosaccharides and linkages as nodes. Then, a series of graph convolutions is performed, in which each node is described by its neighbors and where each subsequent convolution defines a wider circle of “neighboring” nodes. This information is then summarized via various pooling operations and further processed until a prediction is returned. Operations such as transforming a glycan into a graph and other processing operations have recently been added to our Python package glycowork,^[^ ^19^ ^]^ which facilitates this and other applications.

Given the obvious benefits of a model predicting protein–glycan interactions, this challenging task is a valuable application of models such as SweetNet. Previous efforts in predicting lectin–glycan interaction perform reasonably well in recapitulating already‐determined experimental data^[^ ^20^, ^21^ ^]^ but lack scalability and the possibility of providing predictions for novel lectins, which is essential for making more glycan‐binding proteins available and thus empowering future investigations. Further, previous approaches have, at least in part, lacked model interpretation—learning what the model learned—as well as model application to understanding the manifold roles of lectins in biology.

Therefore, we here propose a new model architecture for lectin–glycan interaction prediction that uses information from both glycans and lectins to be fully generalizable. For this, we use our deep learning glycan model SweetNet, designed for accommodating the branched nature of glycans in combination with a transformer‐based model for protein sequences, using the concept of attention to focus on learned relevant parts of the sequence for prediction. Our resulting model, LectinOracle, accurately predicts Z‐score transformed relative fluorescence units for lectin–glycan interactions, showing significant correlation with data from various custom glycan arrays not included during training and agreement with literature on newly characterized lectins. Based on predicted glycan‐binding specificity of characterized and uncharacterized lectins, we suggest a lectin classification system–aided by sugar specificity in addition to protein folds—that spans taxonomic groups, simplifying the task of selecting suitable lectins for experimental purposes. By providing open access to LectinOracle and demonstrating its utility in a wide range of applications, we aim to provide necessary tools for expanding our understanding of lectin–glycan interactions in diverse topics such as disease susceptibility, host–microbe interactions, agriculture, and (auto)immune disease.

## 2 Results

### 2.1 Developing a Deep Learning‐Based Model to Predict Lectin–Glycan Interactions

**Figure 1:**
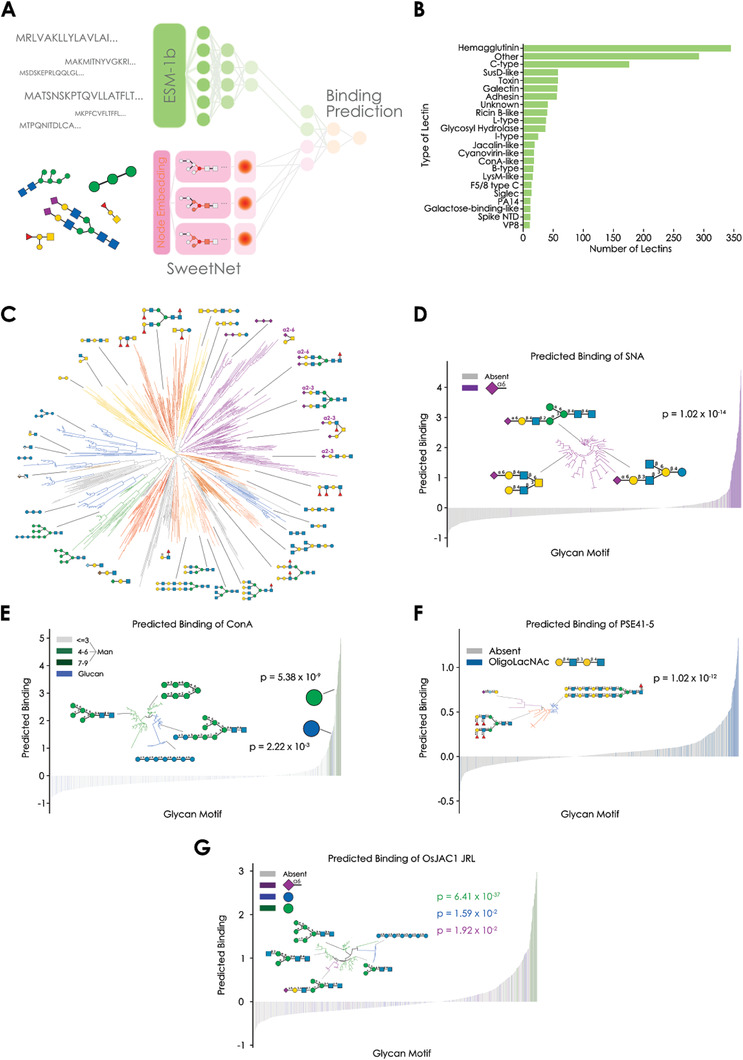
Predicting lectin binding specificity with deep learning. A) Scheme of the deep learning model LectinOracle, which analyzes protein sequences via a pre‐trained transformer‐based model (ESM‐1b) that is further fine‐tuned, and glycan sequences via a graph convolutional neural network (SweetNet). Results from both arms of the model are concatenated and used to predict lectin–glycan binding. B) Data set composition. Shown is the composition of our lectin–glycan data set for training LectinOracle, with the frequency of each lectin class depicted. Siglecs have been highlighted separately from other I‐type lectins due to their biological relevance. C) Glycan clusters in data set. The diversity and relatedness of glycans in our data set is shown via a dendrogram obtained by neighbor joining of the representations learned by the SweetNet component of LectinOracle. The dendrogram is visualized via the Interactive Tree of Life v5.5 software^[^ ^23^ ^]^ and annotated with glycan groups. D,E) Analysis of known lectins via LectinOracle, with the example of SNA and ConA, respectively. For a range of glycan motifs, a trained LectinOracle model was used to predict their binding to the lectins SNA (D) or ConA (E), with their literature‐annotated binding motifs colored. Analysis of uncharacterized lectins, with the example of F) PSE41‐5 and G) the jacalin‐related domain from OsJAC1. Motifs which were identified to be enriched in predicted bound glycans are colored. Motif enrichment was tested via one‐sided Wilcoxon signed‐rank tests, with the *p*‐value shown in each panel. Glycans predicted to be bound were also visualized via dendrograms similar to (C).

To develop a model to predict and analyze protein–glycan interactions, we reasoned a setup was necessary that considered both protein and glycan information to arrive at a binding prediction. This was based on our vision to use the resulting model to extrapolate to interactions of new lectins as well as new glycans. We used protein sequences as inputs for our model rather than protein structures, as the amount of data available for the former far surpasses the latter. Importantly, sequence‐based data contain evidence for glycans and proteins that do not interact, a crucial type of data that is lacking from protein–glycan co‐crystallizations, which only depict successful interactions. Further, protein–glycan co‐crystallizations typically contain only very short glycan fragments, represent nonquantitative data, and, due to the molar excess of glycan fragments during crystallization and the absence of competing glycans, might not constitute physiologically relevant interactions. Finally, working with sequences also ensured that our model can readily be extended to uncharacterized lectins, as sequences are substantially easier to acquire than structures. To train such a model, we constructed a comprehensive data set of 564647 unique protein–glycan interactions from 3328 glycan microarray experiments (Tables S1 and S2, Supporting Information), from the Consortium for Functional Glycomics as well as the Carbohydrate Microarray Facility of Imperial College London. Our data set consisted of 1392 lectins, including plant, fungal, bacterial, viral, and animal lectins (**Figure** **1**B), as well as 927 glycans (Figure 1C). On these data, we trained deep learning‐based models, predicting Z‐score transformed relative fluorescence units (representing binding) based on protein and glycan sequences.

To analyze glycan sequences, we used the state‐of‐the‐art SweetNet architecture, as it has been shown to generally outperform alternative methods and to efficiently scale with glycan diversity.^[^ ^8^ ^]^ SweetNet comprises a graph convolutional neural network that was designed to accommodate the branching structures of glycans. In general, a deep learning‐based glycan analysis module learns similarities between glycan motifs and can more easily generalize to new motifs than a discretized, motif counting‐based approach that is more suited to data analysis than prediction.^[^ ^17^ ^]^

For analyzing protein sequences, we evaluated different approaches. Previous work used recurrent neural networks (RNNs), a type of language model, to predict the interaction of viral hemagglutinin proteins with glycans,^[^ ^8^ ^]^ analyzing receptors for influenza virus. Yet the analysis of protein sequences via deep learning has greatly progressed and transformer‐based models have been shown to outpace RNNs for purposes such as predicting protein function.^[^ ^16^, ^22^ ^]^ Therefore, we chose protein representations learned by ESM‐1b,^[^ ^16^ ^]^ a 650 million parameter model trained on the entirety of UniRef50, as the input for our model, so that this rich representation could be further fine‐tuned for the task presented here.

After processing both protein and glycan sequences, our model concatenated representations learned for both interaction partners and used this information to predict protein–glycan binding via a fully connected neural network module. We evaluated different variations of this model scheme (**Table** **1**) and concluded that the variant with a fine‐tuned ESM‐1b module for protein sequences, a SweetNet‐based module for glycan sequences, and a subsequent fully connected neural network with multisample dropout and sigmoid output scaling resulted in the best empirical performance. We therefore based all further analyses on this model, which we have named LectinOracle (Figure 1A), that has been trained on a wide range of different lectin classes (Figure 1B).

**Table 1:** Selecting an architecture for a model predicting protein–glycan interactions. For the task of predicting protein–glycan interactions, we trained deep learning models with varying architectures to identify a suitable model for this study. In this table, we note the differences in the various models in three modules, the arm analyzing protein sequences, the arm analyzing glycan sequences, and the downstream module combining protein and glycan information for binding prediction. Mean values from five independent training runs (Table S3, Supporting Information, *n* = 5) are provided for the mean squared error (MSE) and mean absolute error (MAE) on a separate test set. For each metric, the best value is shown in bold

Protein arm	Glycan arm	Combined head	MSE	MAE
RNN	SweetNet	Fully connected + Multi‐sample dropout + Sigmoid	0.9925	0.501
ESM‐1b	SweetNet	Fully connected + Multi‐sample dropout + Sigmoid	0.7475	0.4276
ESM‐1b + fine‐tune	SweetNet	Fully connected + Sigmoid	0.7375	0.4238
ESM‐1b + fine‐tune	SweetNet	Fully connected + Multi‐sample dropout	0.7415	0.4301
ESM‐1b + fine‐tune	SweetNet	Fully connected + Multi‐sample dropout + Sigmoid	**0.7283**	**0.4137**

Next to predicting protein–glycan interactions, a trained LectinOracle model can also be used to retrieve representations—learned similarities—for both proteins and glycans. These representations can be used to cluster sequences, such as the glycan sequences in our data set (Figure 1C). Importantly, “similarities” are task‐dependent and therefore should, in this case, reflect similarities in binding behavior. While tasks such as predicting the taxonomic origin of a glycan typically result in glycan representations clustered by class (*N*‐linked, *O*‐linked, etc.),^[^ ^8^, ^17^ ^]^ here we observe a clustering that spans classes and is largely influenced by terminal glycan motifs (e.g., *α*2‐3 linked Neu5Ac, *α*2‐6 linked Neu5Ac, terminal GalNAc). As these terminal motifs are crucial for determining lectin‐binding,^[^ ^24^ ^]^ we concluded that LectinOracle seemed to have learned to extract relevant information from glycans to predict protein–glycan interactions.

We then evaluated the performance of a trained LectinOracle model by analyzing predictions for well‐characterized lectins. First, we chose the lectin *Sambucus nigra* agglutinin (SNA), as it has a well‐defined binding specificity for *α*2‐6 linked Neu5Ac.^[^ ^25^ ^]^ For a range of 1578 glycan motifs occurring in our data set (see the Experimental Section for details), we retrieved binding predictions for SNA from LectinOracle (Figure 1D). Among the predictions, we observed a striking enrichment for *α*2‐6 linked Neu5Ac‐containing motifs, which was highly significant based on a Wilcoxon signed‐rank test (*p* = 5.38 × 10^−9^). In contrast to the highly specific binding specificity of SNA, we investigated the binding behavior of *Aleuria aurantia* lectin (AAL) and confirmed the broad recognition of fucose residues^[^ ^26^ ^]^ in various linkages (Figure S1A, Supporting Information). We additionally analyzed the lectins soybean agglutinin (SBA), from *Glycine max*, and *Helix pomatia* agglutinin (HPA) and demonstrated that LectinOracle correctly learned their preference for terminal GalNAc residues^[^ ^27^, ^28^ ^]^ (Figure S1B,C, Supporting Information).

Next, we analyzed Concanavalin A (ConA), from the jack‐bean *Canavalia ensiformis*, which is known to bind to mannose‐rich glycans.^[^ ^29^ ^]^ We observed an overwhelming enrichment of high‐mannose structures in motifs that were predicted to be bound by ConA (Figure 1E), again confirming its literature‐annotated binding specificity. Additionally, we identified a weaker binding preference for glucan motifs, which is consistent with reports that ConA can bind glucose‐rich sequences,^[^ ^30^ ^]^ such as from fungi. This clear separation between dominant (mannose‐rich) and secondary (glucose‐rich) binding motif that we observed for ConA suggested that the absolute predicted binding by LectinOracle scales, to a certain extent, with affinity or binding strength, which we also explored further in later sections. We indeed observed that, for both SNA and ConA, the binding prediction, on average, increased with a higher number of binding motifs in a glycan (Figure S2, Supporting Information).

We also leveraged the generalizability of LectinOracle to investigate predicted binding motifs for lectins that are only coarsely characterized, or even entirely uncharacterized. One example for this is the lectin PSE41‐5, identified by reverse vaccinology from *Pseudomonas aeruginosa*,^[^ ^31^ ^]^ which has been shown to bind to terminal beta‐linked galactose. With LectinOracle, we confirmed that type II LacNAc structures, with a terminal beta‐linked galactose, were strongly enriched among the predicted binding motifs (Figure 1F). Applied to the relatively uncharacterized jacalin‐related domain from OsJAC1, a lectin from the important food crop *Oryza sativa* (Asian rice), LectinOracle predicted binding predominantly to mannose‐containing motifs, yet also secondary binding to glucose and, specifically, Neu5Ac(*α*2‐6)‐containing motifs (Figure 1G). Recent studies with mono‐ and disaccharides have indeed shown binding to mannose and glucose for this domain,^[^ ^32^ ^]^ whereas sialic acid was not tested for binding. Interestingly, the original jacalin, derived from jackfruit, has been shown to be capable of binding to Neu5Ac,^[^ ^33^ ^]^ lending further support to our analyses.

We also predicted the binding specificity of OTV1_139, a hypothetical protein from *Ostreococcus tauri* virus 1^[^ ^34^ ^]^ that contains a carbohydrate‐binding module 47 (CBM47) domain, which has been shown to bind fucose.^[^ ^35^ ^]^ LectinOracle, applied to OTV1_139, also revealed a preference for binding fucose (Figure S1D, Supporting Information). We next investigated two more lectins, *Arundo donax* lectin (ADL) and hypothetical cytosolic protein 031524 from *Bacillus subtilis* (YesU). For ADL, LectinOracle correctly inferred the activity of its chitin‐binding domain^[^ ^36^ ^]^ and predicted motifs such as chitotriose as the top binders (Figure S1E, Supporting Information). YesU has been characterized to prefer fucosylated glycans^[^ ^37^ ^]^ and LectinOracle also predicted fucosylated glycan motifs to be strongly enriched for binding (Figure S1F, Supporting Information), including fucosylated motifs such as Lewis X. These case studies involving lectins outside our data set emphasize that LectinOracle can be used generally to further probe the glycan‐binding specificity of lectins, both already characterized as well as uncharacterized.

We next engaged in a more comprehensive validation of lectin specificity prediction. For this, we compared LectinOracle predictions to the in‐depth lectin annotations recently made via a combination of machine learning and manual expert annotation of 57 commonly used lectins.^[^ ^38^ ^]^ For the 51 lectins for which we could retrieve an amino acid sequence, LectinOracle identified the correct binding motif in over 90% of lectins (Figure S3A, Supporting Information), even though 19/51 lectins were not part of our training set and therefore entirely new to LectinOracle.

We then compared LectinOracle to other existing approaches that, at least in part, have a similar goal related to understanding lectin–glycan binding, such as GlyNet.^[^ ^21^ ^]^ Testing GlyNet on these 51 lectins revealed an accurate prediction for 40/51 lectins (≈78%; Figure S3B, Supporting Information). This lower result, despite the fact that GlyNet was trained on all tested lectins, indicates that LectinOracle has a higher accuracy in predicting lectin binding specificity. Additionally, other approaches, such as GlyNet, cannot use protein information provided by, for instance, the ESM‐1b representations in this study. This constitutes a unique feature and definite advantage of LectinOracle, both in terms of performance as well as generalizability. It also enabled the large‐scale applications with uncharacterized lectins described below that would be impossible with existing methods. Further, compared to approaches predicting monosaccharide specificity based on lectin classes or folds, LectinOracle can predict the binding of any lectin to glycan motifs of arbitrary complexity, making our approach qualitatively different from existing approaches.

### 2.2 Analyzing Lectins with LectinOracle Reveals Lectin Clusters with Shared Binding Patterns

The categorization of lectins into different classes, such as galectins^[^ ^39^ ^]^ or LysM‐like lectins,^[^ ^40^ ^]^ has aided the understanding of lectin interactions on a systematic level. Often, lectins are categorized based on shared structural characteristics, such as a common fold.^[^ ^11^, ^41^ ^]^ However, structural similarity does not always lead to similar overall binding preferences and distinct protein folds, with shared binding specificity, have evolved independently.^[^ ^6^ ^]^ We hypothesized that a lectin classification that also factored in (predicted) binding specificity would improve on this structural approach and lead to classes that would be immediately useful to researchers who use lectins in their work and rely on their binding specificity rather than their structure.

**Figure 2:**
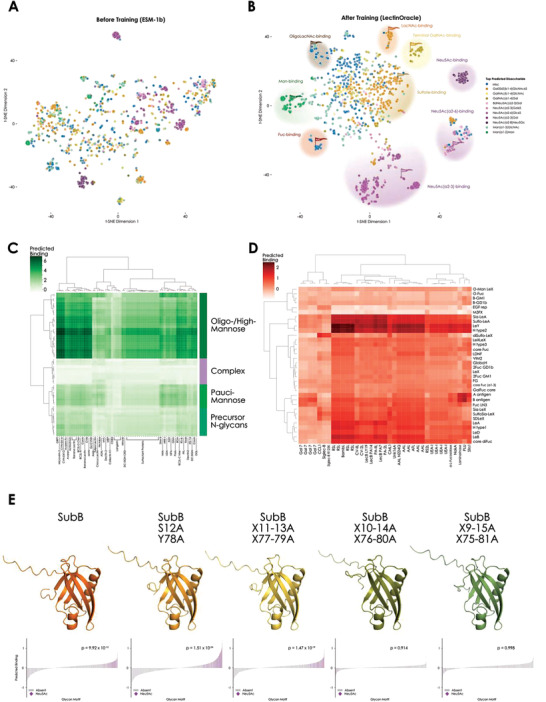
Clustering lectins based on learned binding motifs. A,B) Lectins clustered based on sequence similarity or binding specificity. Learned representations from the pre‐trained ESM‐1b model (A) or a trained LectinOracle model (B) were extracted for each lectin and are shown via t‐SNE,^[^ ^45^ ^]^ colored by the top predicted disaccharide binding motif. Cluster binding specificities are annotated and representative lectins are labeled. C) Characterization of mannose‐binding lectins. We selected the group of lectins with “Man(*α*1‐2)Man” as the top‐predicted disaccharide and used LectinOracle to predict their binding to a range of mannose‐containing glycans. Then, we performed hierarchical clustering using Ward's method.^[^ ^46^ ^]^ D) Characterization of fucose‐binding lectins. After selecting lectins with fucose within their top predicted disaccharide, we predicted their binding to a range of fucose‐containing motifs from the academic literature and filtered out lectins which did not have at least a predicted binding of one to any of these motifs, depicting the rest as a hierarchical clustering based on Ward's method. E) LectinOracle predictions are sensitive to amino acid substitutions in the lectin binding pocket. For SubB and various alanine‐substitution mutants, we predicted their glycan‐binding behavior with a range of glycan motifs. One‐sided Wilcoxon signed‐rank tests were used to ascertain significant enrichment for predicted Neu5Ac binding in each case. Protein structure predictions made with AlphaFold2 are shown for the wild‐type protein and each mutant.

When coloring lectins based on the disaccharide that is the top prediction from LectinOracle, we overall did not observe meaningful binding specificity clustering in the ESM‐1b representation, that solely relies on sequence similarity (**Figure** **2**A). Possible exceptions to this were Neu5Ac‐binding lectins that exhibited high sequence similarity and were prevalent in our data set, such as the influenza virus hemagglutinins.^[^ ^42^ ^]^ However, once we plotted the lectin representations learned by LectinOracle (sequence similarity + binding specificity), we immediately noticed several distinct lectin clusters that seemed to share binding patterns (Figure 2B). Clustering by lectin characteristics other than sequence similarity might also result in informative clusters. However, lectin annotation and documentation are incomplete, which is the reason for the existence and relevance of UniLectin3D.^[^ ^43^ ^]^ To partially overcome this, we used NetGO 2.0^[^ ^44^ ^]^ to predict GO term annotations for all our lectins and used this for clustering (Figure S4, Supporting Information). Similar to sequence similarity, predicted GO terms did not lead to an overall meaningful clustering in terms of binding behavior, re‐emphasizing the added value of representations learned by LectinOracle for this purpose.

Most strikingly, we observed a mannose‐binding lectin cluster that was not apparent in the clustering based on sequence similarity. This cluster contained well‐known mannose‐binding lectins, such as plant‐derived ConA and the Banana lectin from *Musa acuminata* as well as mammalian lectins, such as the human Mannose Binding Lectin 2 (MBL2). While most clusters exhibited a dominant disaccharide that characterized cluster binding, fucose‐binding lectins demonstrated a pronounced diversity in their exact fucose specificity between lectins, with no single overarching disaccharide despite forming a cluster in our representation. With regards to sialic acid‐binding, we could annotate multiple clusters, with specificity for *α*2‐3 linked Neu5Ac, *α*2‐6 linked Neu5Ac, and Neu5Ac without linkage‐preference, respectively. These clusters contained the expected avian (*α*2‐3 linked Neu5Ac) as well as mammalian influenza virus hemagglutinins (*α*2‐6 linked Neu5Ac).^[^ ^47^ ^]^ Yet we also identified other lectins in these clusters that were less obvious via sequence similarity, such as *Staphylococcus aureus* superantigen‐like 6 (SSL6) or *Escherichia coli* heat‐labile enterotoxin for the *α*2‐3 linked Neu5Ac cluster and SNA or *Polyporus squamosus* lectin (PSL) in the *α*2‐6 linked Neu5Ac cluster. This clustering beyond mere sequence similarity, which was enabled by LectinOracle, holds promise for re‐classifying existing lectins as well as annotating and discovering new lectins that could be used as research probes or shed light on biological phenomena.

Next, we sought to further characterize the clusters unveiled by the similarities learned by LectinOracle (Figure 2B) and thereby propose a more useful characterization of lectins, not only informed by sequence similarity but additionally by binding specificity. We first analyzed the cluster containing mannose‐binding lectins, which not only cleanly separated different classes of mannose‐containing glycans based on their predicted binding but also revealed lectin subgroups in this cluster that exhibited slightly different binding preferences (Figure 2C). One cluster, containing cyanovirin‐like lectins, such as microvirin or cyanovirin, as well as the jacalin‐like banana lectin, was predicted to bind especially well to oligo‐ and high‐mannose glycans. Similar to a dose‐response relationship, lectins with a higher predicted binding to oligo‐/high‐mannose structures also exhibited, albeit lower, residual predicted binding to pauci‐mannose structures (3–4 mannoses). This high‐level characterization might serve as a guideline for researchers choosing a lectin that is best suited to their experimental needs.

Analogously, we analyzed fucose‐binding lectins, based on widely known fucose‐containing motifs from the academic literature (Figure 2D). Our first observation was that, while galectin 7 and siglec 8 were filtered together with the fucose‐binding lectins, the motif heatmap revealed that they were only predicted to substantially bind to sialylated/sulfated motifs (siglec 8) or motifs containing LacNAc structures (galectin 7), not to other fucosylated structures. The remaining block of lectins represented the “true” fucose‐specific lectins. Here, we also observed a dose response, in that difucosylated motifs (Lewis B, Lewis Y, difucosylated N‐glycan cores, etc.) were, on average, predicted to be bound stronger than monofucosylated motifs. With analyses such as these, our platform constitutes a rapid means to characterize and compare a large set of lectins with regards to their binding specificity to identify subclusters as well as the most appropriate lectin reagents for detecting glycan motifs.

We were also intrigued to observe two distinct clusters for lectins predicted to bind the N‐acetyllactosamine (LacNAc) motif that is near‐ubiquitous in animals (Figure 2B; light brown and dark brown). We therefore decided to identify what distinguished lectins from both clusters. As type I and type II LacNAc can occur in multiple forms (LacNAc, OligoLacNAc, PolyLacNAc), we used our trained LectinOracle model to predict the binding of all lectins in our data set to one, two, or three repeats of type II LacNAc (Gal(*β*1‐4)GlcNAc(*β*1‐3); Figure S5, Supporting Information). When coloring all lectins according to their predicted binding, we observed that one of the two clusters started out with strong predicted binding to 1xLacNAc yet seemed to lose predicted binding with 3xLacNAc. This cluster was enriched in epithelial adhesins from the fungus *Candida glabrata*.^[^ ^48^ ^]^ The other cluster, however, increased in predicted binding with increasing number of LacNAc repeats. Here, we found an enrichment for galectins from various species. This led us to the conclusion that the first cluster constituted LacNAc‐binders while the second cluster was rather characterized by oligoLacNAc‐binding. This also implied that LectinOracle was sensitive to the number of repeats for a glycan motif, which we already indicated with the example of SNA and ConA above (Figure S2, Supporting Information).

Beyond short di‐ or trisaccharide motifs, many larger motifs, such as the Lewis structures,^[^ ^49^ ^]^ are widely known and relevant in various biological contexts. We therefore engaged in an analysis to find out whether we could identify clusters of lectins binding to these motifs (Figure S6, Supporting Information). While we could pinpoint distinct regions for many motifs in the lectin space learned by LectinOracle, clustering in most cases could be explained by smaller motifs within larger motifs such as the Lewis series. The presence of fucose in Lewis structures and blood group epitopes led to activity in the fucose‐binding cluster, motifs such as SialylLewis X added predicted binding from Neu5Ac‐binding lectins, and the blood group A antigen included activity from the terminal GalNAc‐binding cluster. Antibodies with high specificity for a defined large motif notwithstanding, most lectins in our data set seem to be well‐characterized on the disaccharide level.

Having established the sensitivity of LectinOracle to minute, monosaccharide‐level changes in glycans, we then set out to analyze model sensitivity toward the lectin sequence. In other words, did LectinOracle learn a broader domain—motif relationship or a more fine‐grained sequence/binding pocket—motif association? For this, we turned to *E. coli* Subtilase cytotoxin subunit B (SubB), a protein outside our data set and part of a bacterial AB5 toxin that binds sialic acids with two key amino acid residues, S12 and Y78.^[^ ^50^ ^]^ We analyzed the glycan binding of SubB with LectinOracle and indeed observed significant enrichment of Neu5Gc‐ and Neu5Ac‐containing moieties among the predicted binding glycans (Figure 2E and Figure S7, Supporting Information). We note that, while we did observe a statistically significant binding prediction for Neu5Gc binding (Figure S7, Supporting Information), due to a relative scarcity of Neu5Gc‐containing glycan motifs on the arrays used for training LectinOracle we were unable to quantify any differential preference of Neu5Gc over Neu5Ac. Interestingly, we found that the binding predictions for SubB are highly sensitive toward amino acid substitutions in the glycan‐interacting region of the protein, with Neu5Ac/Gc enrichment being completely abolished upon substituting five residues around each key amino acid with alanine (Figure 2E and Figure S7, Supporting Information), while the substitutions did not substantially impact the protein structure as predicted by AlphaFold2.^[^ ^14^ ^]^ Overall, these results demonstrate that LectinOracle, without any prior knowledge or being trained for this, is sensitive toward mutations of amino acids in the ligand binding pocket of SubB. However, it is still unclear how sensitive LectinOracle is to single point mutations versus critical sequence stretches, as the mutation of the two key binding residues itself did not lead to an abrogation of predicted binding. In fact, even large‐scale models such as AlphaFold2 were recently shown to be not sensitive toward single point mutations,^[^ ^51^ ^]^ indicating that this might require dedicated training.

Having established the sensitivity of LectinOracle to critical amino acid stretches for binding predictions, we next set out to investigate whether our model could be used in the context of lectin directed evolution. Screening a large set of mutants can be used to shift lectin binding specificity, for instance, demonstrated in a study evolving the C‐terminal domain of the EW29 lectin (EW29Ch) from the earthworm *Lumbricus terrestris* toward a 6′‐sulfo‐galactose binding lectin.^[^ ^52^ ^]^ Predictions from LectinOracle for the mutants from this experiment matched experimental results, where mutants with a higher predicted binding also showed higher binding to 6′‐sulfo‐LacNAc (Gal6S(*β*1‐4)GlcNAc) and neutral mutants having no change in their predicted binding (Figure S8, Supporting Information). This proof‐of‐concept analysis demonstrates the potential of LectinOracle to be used to accelerate lectin directed evolution approaches.

### 2.3 LectinOracle Predictions Match Independent Experimental Observations Qualitatively and Quantitatively

The gold standard for validating machine learning models is to compare their predictions to independent experimental observations. Not only does this procedure allow for the evaluation of the quality of a model but it also showcases the range of scenarios where a model can be applied with sufficient accuracy, as model predictions typically deteriorate the further a scenario is removed from the training data.

As a first validation, we compared our predictions to co‐crystallized lectin–glycan structures found in UniLectin3D.^[^ ^43^ ^]^ For this, we used the lectin and the glycan in the complex as input to LectinOracle to see whether this binding would have been predicted. For lectins that were part of our training set, LectinOracle achieved good predictive accuracy for lectins from all taxonomic groups (Figure S9A, Supporting Information) and nearly all lectin classes (Figure S9B, Supporting Information). When including all lectins, we did observe lower performance for archaeal lectins (absent from our data set) and viral lectins (Figure S9C,D, Supporting Information). As mentioned before, while offering valuable mechanistic insights into protein–glycan interactions, co‐crystallized lectin–glycan structures should not be seen as the gold standard of predicting lectin–glycan binding due to potential limitations. We were nonetheless satisfied that LectinOracle, on average, predicted the interactions of the majority of lectins on UniLectin3D.

**Figure 3:**
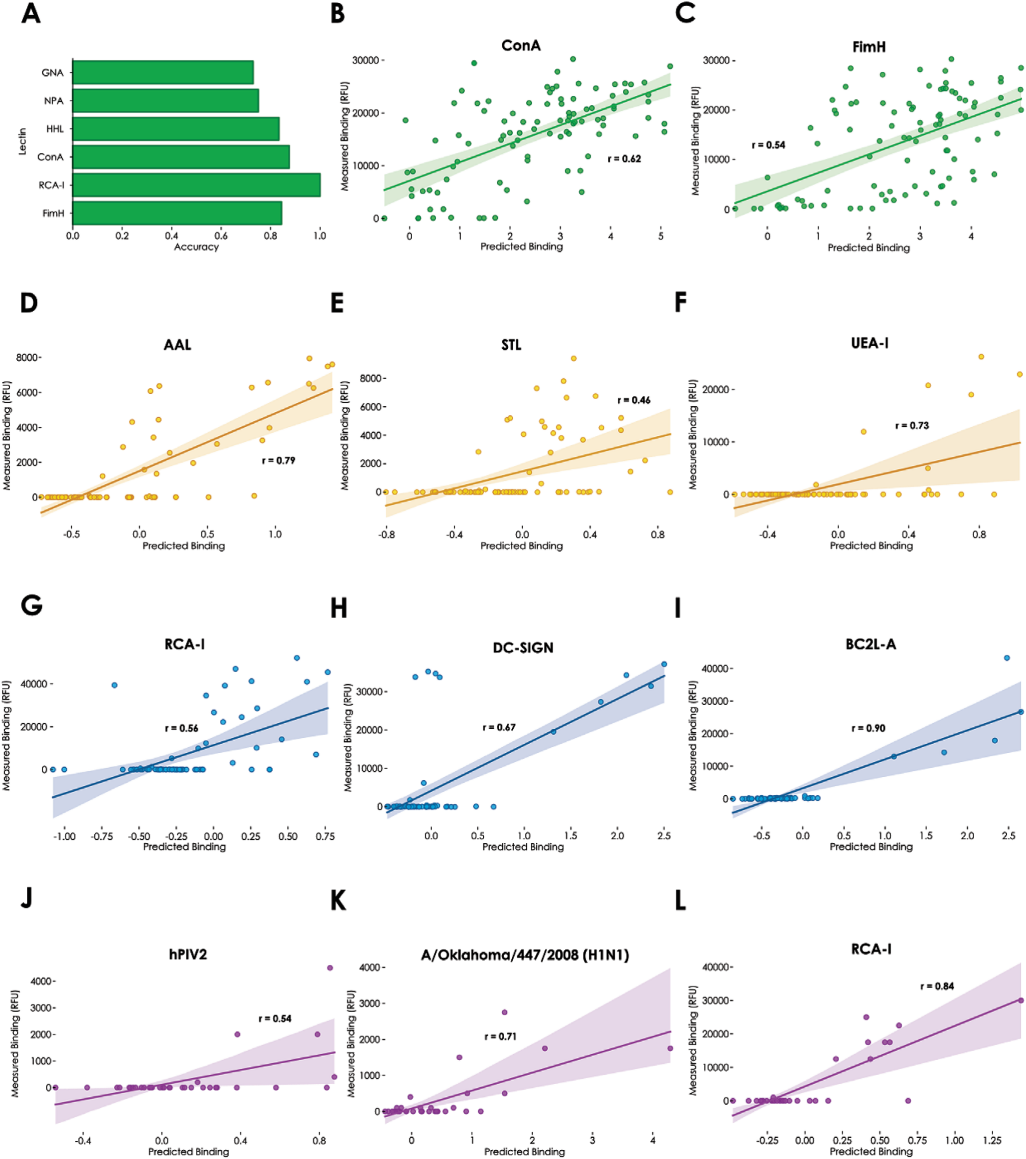
LectinOracle predicts binding of lectins to a wide range of different glycan arrays. A) Accuracy of LectinOracle predictions for lectins tested on the oligomannose array. For each lectin–glycan pair, we assigned it the label “bound” or “predicted bound” if the observed relative fluorescence units (RFU) were at least 10% of the maximum RFU or if the predicted binding was at least 0.5, respectively. The agreement of experiment and prediction is shown in terms of accuracy (precision can be found in Figure S10A, Supporting Information). Correlating experimentally observed binding with predictions for the B) lectins ConA and C) FimH tested on the oligomannose array. D–F) Correlating experimentally observed binding with predictions for the lectins AAL, STL, and UEA‐I, respectively, on the mucin *O*‐glycan array. G–I) Correlating experimentally observed binding with predictions for the lectins RCA‐I, DC‐SIGN, and BC2L‐A, respectively, on the microbe‐focused array. J–L) Correlating experimentally observed binding with predictions for the lectins hPIV2, A/Oklahoma/447/2008 (H1N1), and RCA‐I, respectively, on the sialic acid array. All correlations between experimental data and predictions were done via fitting a linear regression and *r* represents Pearson's correlation coefficient.

To circumvent potential issues of crystal structures, we also used experimental data from a range of customized glycan arrays that were not part of the CFG or Imperial College database to validate LectinOracle. These arrays contained glycans as well as lectins that were new to LectinOracle and offered a rich source for validating our predictions. First, we used the recently published oligomannose array for this purpose.^[^ ^53^ ^]^ Here, investigators assayed a variety of subtly different oligomannose glycans for their binding to various lectins. The slight changes in glycan structure, which nonetheless led to appreciable differences in their binding behavior, made this a particularly challenging case study for our model. When thresholding our predictions and the observed relative fluorescence units (i.e., separating glycans into “bound”/“not bound” for a given lectin), we achieved predictive accuracies of ≈70–90% for plant and bacterial lectins using LectinOracle (**Figure** **3**A and Figure S10A, Supporting Information). With the exception of Dectin‐2, we observed similar results for mammalian lectins on this array (Figure S9B–D, Supporting Information). We note that for the antibodies tested on this array, LectinOracle did not yield correct predictions (Figure S10C,D, Supporting Information). This is likely due to two factors: i) the antibodies are highly specific, with a low single‐digit number of bound glycans on the oligomannose array that are easier to miss entirely and ii) our training set lacks data for antibodies, so LectinOracle was never trained to be able to predict antibody‐glycan binding and should not be used for this purpose.

Encouraged by these results, we hypothesized that the relative predicted binding from LectinOracle could be informative beyond classifying glycans into “bound”/“not bound,” as LectinOracle was trained on quantitative binding data. Therefore, for each glycan, we correlated predicted binding and experimentally measured binding for a range of lectins (Figure 3B,C and Figure S10B, Supporting Information), to see whether we could predict quantitative binding, a substantially more challenging task. For lectins on the oligomannose array, we indeed achieved moderate correlations (defined as a Pearson's correlation coefficient between 0.5 and 0.7) between our predictions and the experimental results, demonstrating that our predictions contain quantitative information about protein–glycan interactions, even in a different array setting than LectinOracle was trained on.

To show that this constitutes a general property of LectinOracle and to test the generalizability of its predictions in different contexts, we then went on to validate our predictions on other glycan arrays. First, we tested the recently reported mucin *O*‐glycan array,^[^ ^54^ ^]^ in which 83 *O*‐linked glycans with various modifications were tested against several lectins. In this context, with very different glycans compared to the oligomannose array, LectinOracle again achieved a quantitative correlation to independent experimental data (Figure 3D–F), with some lectins, such as AAL and UEA‐I, even showing a strong correlation between predictions and experimental observations (Pearson's correlation coefficient > 0.7). We extended this to two additional array types, the microbe‐focused glycan array^[^ ^55^ ^]^ (Figure 3G–I) and the sialic acid array^[^ ^56^ ^]^ (Figure 3J–L). In both cases, we observed moderate to strong correlations between the predicted binding values from LectinOracle and the experimentally measured binding.

In some cases, such as DC‐SIGN on the microbe‐focused array (Figure 3H), LectinOracle correctly predicted the binding to one cluster of glycan sequences (mannose‐rich glycans) yet missed another cluster of glycans in its predictions that was measured to bind (Lewis X‐type structures^[^ ^57^ ^]^). To better understand this misprediction of Lewis X‐type (LeX) structures, we investigated the binding behavior of DC‐SIGN in depth (Figure S11, Supporting Information). To our surprise, we found more non‐binding than binding LeX‐containing glycans in the DC‐SIGN glycan array data. From our analyses, it seemed that only terminal LeX structures, with Fuc(*α*1‐2) as the only permitted extension, had a chance to be bound by DC‐SIGN and, even in those cases, sialylation (even on a different antenna) strongly inhibited binding, such as in the case of Fuc(*α*1‐2)Gal(*β*1‐4)[Fuc(*α*1‐3)]GlcNAc(*β*1‐3)[Neu5Ac(*α*2‐6)Gal(*β*1‐4)GlcNAc(*β*1‐6)]Gal(*β*1‐4)Glc, which showed absolutely no binding to DC‐SIGN, while Fuc(*α*1‐2)Gal(*β*1‐4)[Fuc(*α*1‐3)]GlcNAc(*β*1‐3)Gal(*β*1‐4)[Fuc(*α*1‐3)]GlcNAc represented the strongest binder on the array. Overall, this suggests a complex binding behavior between DC‐SIGN and LeX, that, to our knowledge, seems to have even been overlooked by in‐depth expert annotation until now. We assume that this also makes it more challenging for models such as LectinOracle to learn these somewhat conflicting relationships.

This complexity suggests that, with more data from more diverse glycan arrays, LectinOracle could be further improved to detect all binding specificities of a lectin. Overall, the example of DC‐SIGN and the high precision of our predictions (Figure S10A,D, Supporting Information) implies that errors in LectinOracle predictions are more likely to be false negatives than false positives, raising the confidence in binding predictions. These validations across multiple independent arrays included bacterial, viral, plant, fungal, and mammalian lectins. Additionally, the majority of glycans in these custom arrays were not present in the CFG and Imperial College arrays used for training LectinOracle. We therefore concluded that LectinOracle can generalize to new lectins, new glycans, and new contexts (e.g., different linkers), as long as they are not too far removed from the training set, such as the antibodies mentioned above.

### 2.4 Investigating Lectomes in Host–Microbe Interactions with LectinOracle Uncovers Shifting Binding Repertoires

Lectins are widespread throughout all kingdoms of life and carry out a panoply of essential functions, from combating pathogens^[^ ^58^ ^]^ to distinguishing self and foreign tissue.^[^ ^59^ ^]^ Yet the set of lectins that have been experimentally characterized to a sufficient degree only represents a sliver of the total set of lectins in nature. Genome annotation efforts, based on sequence similarity to known lectins, have resulted in databases such as LectomeXplore,^[^ ^11^ ^]^ where predicted lectins from thousands of species, as well as their lectin class, are catalogued.

**Figure 4:**
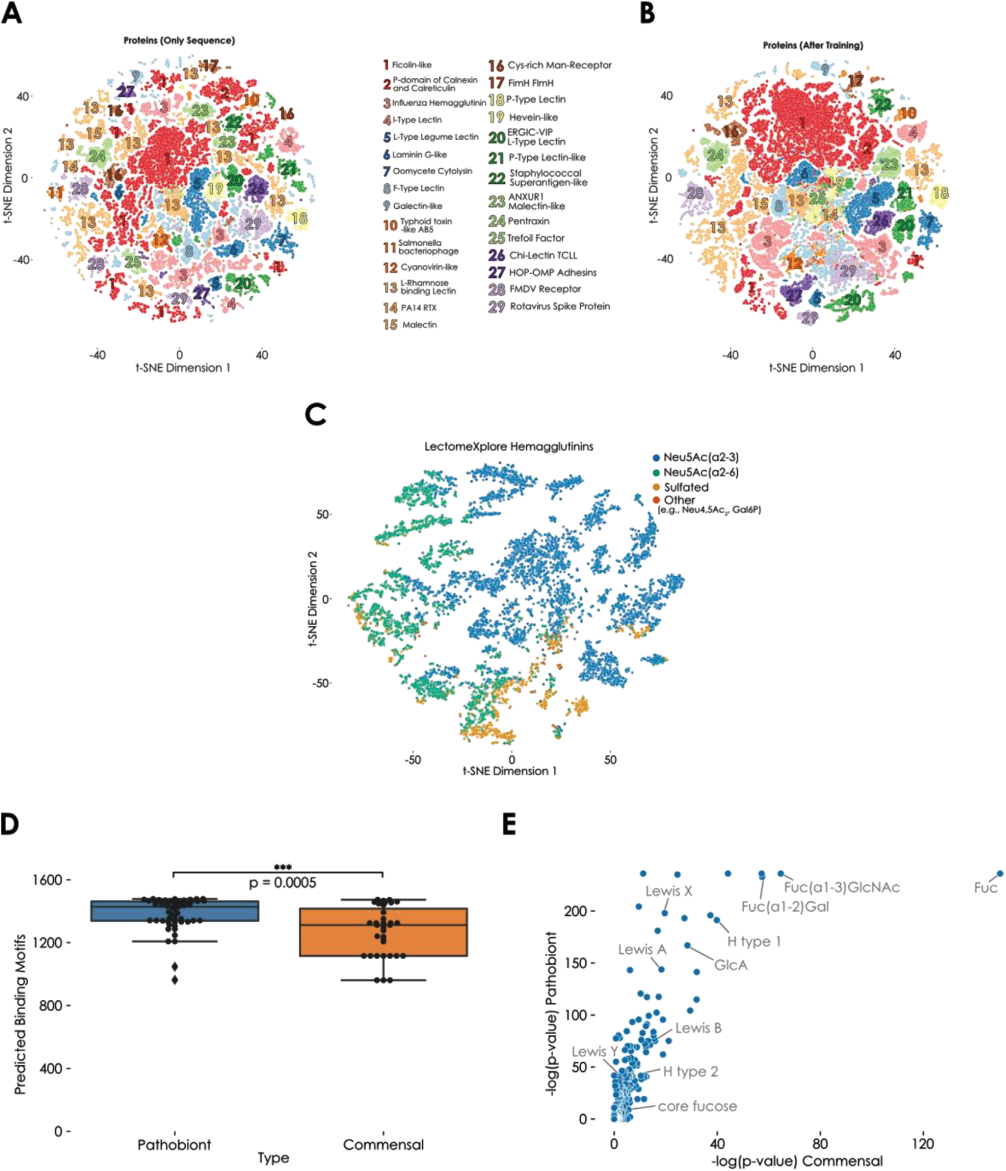
Analyzing lectomes and their role in biology with LectinOracle. A,B) Lectins from LectomeXplore clustered based on sequence similarity or binding specificity. Learned representations from the pre‐trained ESM‐1b model (A) or a trained LectinOracle model (B) were extracted for all 120,523 putative lectins in LectomeXplore with a similarity score higher than 0.5. Lectin classes with at least 400 examples are annotated in both (A) and (B). C) Predicted glycan‐binding specificity for hemagglutinins on LectomeXplore. For 9752 hemagglutinin sequences from LectomeXplore, we plotted their learned representation from a trained LectinOracle model and colored them based on their predicted glycan‐binding specificity. Motifs were colored if they showed a significant enrichment in predicted binding, according to motif‐based one‐tailed Welch's *t*‐tests and a Holm–Šídák correction for multiple testing. D) Pathobionts in the vaginal microbiome have a larger predicted binding repertoire than commensals. For all strains in the data set, we used a trained LectinOracle model to predict the binding motifs of their lectins. The number of motifs with a predicted binding above zero is shown for both pathobionts and commensals as mean ± s.e.m. Statistical significance between groups (pathobionts *n* = 55, commensals *n* = 35) was established with a two‐tailed Welch's *t*‐test. ***p < 0.001. E) Enriched predicted binding motifs for lectins from pathobionts and commensals. For all lectins from pathobionts and commensals, we predicted their binding to a range of glycan motifs and analyzed enriched binding motifs via one‐tailed Welch's *t*‐tests and a Holm‐Šídák correction for multiple testing. The resulting *p*‐values per motif are shown as −log(*p*‐value), with representative motifs annotated.

Seeking to better understand these predicted lectins, we used our LectinOracle platform to analyze lectins in LectomeXplore with a prediction score of 0.5 or above (i.e., at least 50% similarity to known lectins). This corresponded to 120,523 putative lectins from 7753 species, which in turn represented 113,573 unique protein sequences. We calculated ESM‐1b representations for these sequences and observed that, while many assigned lectin classes clustered together based on sequence similarity (**Figure** **4**A), classes such as ficolin‐like lectins or l‐rhamnose binding lectins demonstrated a fragmented cluster behavior, suggesting a sequence diversity or sub‐classes within these categories. It should be noted that broad classes such as ficolin‐like lectins, defined by containing fibrinogen‐like domains, could also contain non‐lectin proteins.

Next, we visualized the learned lectin representations after training LectinOracle (Figure 4B). These representations, factoring in learned glycan‐binding specificity in addition to sequence similarity, yielded an improved clustering, with a lower cluster variance for most lectin classes (Figure S12A, Supporting Information). Classes such as foot‐and‐mouth disease virus (FMDV) receptor lectins, which showed two distinct clusters when clustering by ESM‐1b representations, were unified when clustering on both sequence similarity and binding specificity. We also note that the sequences in a cluster often corresponded to very uniform LectomeXplore score values (Figure S12B,C, Supporting Information), implying that they form pools of sequences that are similar to each other, with a comparable similarity to the profile used for searching for the lectin class in genomes. Some classes, such as endoplasmic reticulum golgi intermediate compartment/vesicular integral proteins (ERGIC VIP) l‐type lectins, formed distinct clusters with different score distributions, suggesting that these clusters correspond to different pools of lectins from the ERGIC VIP l‐type class. This could pave the way for a more fine‐grained classification of lectins that may differ in important aspects.

We then further analyzed additional lectin classes on LectomeXplore. One example of broad interest are hemagglutinin proteins, which are crucial for influenza virus cell entry, as the glycan‐binding specificity of hemagglutinin can determine host range.^[^ ^4^ ^]^ Classically, hemagglutinins are split between binding *α*2‐3‐linked Neu5Ac (avian host) and *α*2‐6‐linked Neu5Ac (mammalian host), with additional reports that sulfated^[^ ^60^ ^]^ and phosphorylated^[^ ^61^ ^]^ glycans may be bound. When analyzing the 9752 hemagglutinin sequences from LectomeXplore with a score above 0.5 via LectinOracle, we could clearly separate hemagglutinins into three broad clusters, corresponding to Neu5Ac(*α*2‐3), Neu5Ac(*α*2‐6), and sulfated glycans as their major binding epitope (Figure 4C). We additionally noted the occurrence of smaller clusters that seemed to prefer binding to phosphorylated glycans or O‐acetylated Neu5Ac (Neu4,5Ac_2_).

Considering the segregation of hemagglutinin sequences by LectinOracle, we next investigated whether our influenza‐related predictions corresponded to epidemiological outcomes. For this, we gathered hemagglutinin sequences from 212 H3N2 influenza strains from Taiwan between 1999 and 2007 which have been shown to vary in their preference for binding to human‐like Neu5Ac(*α*2‐6)‐containing motifs.^[^ ^42^ ^]^ Predicting their binding to this motif via LectinOracle, we also observed variation in predicted binding to Neu5Ac(*α*2‐6)‐containing glycans across the years (Figure S13, Supporting Information). Importantly, the fluctuations in predicted Neu5Ac(*α*2‐6) binding closely matched the trajectory of excess deaths due to H3N2 in Taiwan from 2002 to 2006.^[^ ^62^ ^]^ This correspondence suggests that stronger binding to human‐like Neu5Ac(*α*2‐6) predicted by our model could predict epidemiological outcomes such as excess deaths, showcasing another important use case for LectinOracle.

Further analyses into other LectomeXplore classes, such as clustering staphylococcal superantigen‐like lectins or F‐type lectins into subgroups with different glycan‐binding preferences supported the ability of LectinOracle to further characterize these putative lectins at scale (Figure S14, Supporting Information). These results demonstrate that the lectin annotation pipeline could be extended to also include predicted binding specificity for further insight.

Recent work has focused on analyzing the lectomes of pathobionts and commensals in the vaginal microbiome, finding a higher number of lectins in pathobionts than in commensals.^[^ ^63^ ^]^ Analyzing the respective lectin classes resulted in showing that lectomes from pathobionts seemed to bind to a higher diversity of glycan motifs. Using LectinOracle, we arrived at a similar result in that lectomes from pathobionts were predicted to bind to more glycan motifs than those from commensals (Figure 4D), which might aid pathobionts in adhering more robustly to mucosal surfaces.^[^ ^64^ ^]^

We then set out to capitalize on the predictive capabilities of LectinOracle to investigate which glycan motifs were targeted by pathobionts and commensals, respectively (Figure 4E). Based on our predictions, lectins from both pathobionts and commensals seemed to be particularly enriched in binding to fucose‐containing motifs. In accordance with our earlier analysis (Figure 4A), pathobionts exhibited a greater diversity of highly enriched binding motifs, including various fucosylated motifs that are prevalent in human glycans (Lewis X, Lewis A, H type 1, etc.) and that are known to mediate adhesion of other pathobionts such as *Helicobacter pylori*.^[^ ^65^ ^]^ These structures could thus also be used by the pathobionts of the vaginal microbiome to adhere to mucosal surfaces, showcasing the utility of LectinOracle to yield further insight into biological contexts involving lectins.

## 3 Discussion

If glycosyltransferases and related enzymes could be construed as the “writers” in glycobiology, lectins would represent the “readers” of the glycocode.^[^ ^66^ ^]^ Because glycans dominate the surface area of most cells, protein‐mediated interactions between cells or organisms typically rely on lectins that recognize specific glycan motifs. Yet lectins typically do not follow a strict one protein—one motif correspondence such as zinc finger proteins in DNA recognition.^[^ ^67^ ^]^ Mostly, lectins span the whole range, from a narrowly defined binding specificity, such as SNA, to a broader, more relaxed binding specificity that is still well‐defined, such as in the case of ConA. Knowing which glycan motifs are bound by any given lectin is a nontrivial endeavor and usually entails months or even years of dedicated study. This is a problem both for timely issues, such as ascertaining the glycan‐binding specificity of a pandemic‐causing pathogen,^[^ ^68^ ^]^ as well as for systematic issues, as the high‐throughput characterization of whole lectomes^[^ ^11^ ^]^ is currently infeasible. We view LectinOracle as a means to alleviate these bottlenecks, as well as to further characterize and cluster well‐studied lectins, by obtaining binding predictions for lectin sequences in a rapid and scalable manner.

LectinOracle can be extended to both new lectins and new glycans, paving the way for its integration into the routine study and usage of lectins. Potential applications, as shown here, span the range from in‐depth characterization of the binding profile of individual lectins to the analysis of whole lectomes and their binding profile in health and disease, with potential mechanistic and biomedical implications. In future work, approaches such as presented here could also be combined with the tissue‐specific glycome of a host species^[^ ^69^ ^]^ to identify specific glycan receptors that could be physiologically relevant for the adhesion of a pathogen.

A potential limitation of our work could lie in extreme generalizability. We already mentioned that distinct protein subgroups, such as antibodies, might not be amenable to be used as inputs for LectinOracle if they are currently not represented in the training data. In general, lectins that exhibit multiple binding sites with distinct specificities and/or more heterogeneous binding properties might also be harder to learn for models such as LectinOracle, as for instance seen with DC‐SIGN. Further, while new glycans can be readily used as inputs for LectinOracle, we are currently limited to glycans composed of the monosaccharides used for training LectinOracle. Unseen monosaccharides would not have a learned representation and could, at this stage, not be interpreted by LectinOracle. Fortunately, LectinOracle was trained with a large set of 80 monosaccharides and linkages, which will enable researchers to work with most glycans. The integration of future glycan arrays with even more diverse glycans will further improve this type of generalizability. Once these additional data become available in sufficient quantity, we also anticipate further increases in robustness and performance of a re‐trained version of LectinOracle. One example for this can be seen in the case of Dectin2 that shows only binding to oligomannose glycans (e.g., Man(*α*1‐2)Man(*α*1‐2)Man(*α*1‐6)[Man(*α*1‐3)]Man) on our training set arrays but binds to both oligomannose and *N*‐linked oligomannose glycans (e.g., Man(*α*1‐2)Man(*α*1‐2)Man(*α*1‐3)[Man(*α*1‐3)Man(*α*1‐6)]Man(*β*1‐4)GlcNAc(*β*1‐4)GlcNAc) on the oligomannose array, resulting in poor prediction performance due to array discrepancies (Figure S10C, Supporting Information). Future versions of LectinOracle that are also trained on arrays such as the oligomannose array should improve on these results.

Recent research has shed light on the differences between binding conditions on glycan arrays and physiological presentation of glycans on cells and tissues,^[^ ^70^, ^71^, ^72^, ^73^ ^]^ including issues such as crowding, diversity, and linker properties. Additionally, most glycan arrays currently are biased towards mammalian‐like glycans. For some lectins, binding preferences that were found on glycan arrays could not be recapitulated on tissues and vice versa. As LectinOracle was trained on glycan array data, these limitations could extend to its predictions as well. Another important point to note here is that LectinOracle was trained on glycan array data, which are only semi‐quantitative. Future work should therefore explore training such a model on quantitative binding measurements, such as frontal affinity chromatography,^[^ ^74^ ^]^ once enough data of this type become available. Yet, at this stage, there is by far not enough physiological binding data available to train any kind of model. Further, current expert inferences of binding specificity are also informed by array data and typically do not deviate extensively from physiological behavior. By extension, predictions made with LectinOracle should thus also yield physiologically relevant predictions on average. Additionally, as more structural data of lectin–glycan complexes become available, this type of information could eventually be added as a “third arm” of LectinOracle, further improving prediction results and offering a more direct path to mechanistic interpretation. However, this step should ideally be present as an optional input, to not impede generalizability.

In general, learning protein–glycan interactions from sequences instead of structures allowed us to leverage a vastly larger amount of data. Drawbacks with protein–glycan co‐crystallization data, apart from data scarcity, include the lack of available quantitative binding data and the absence of negative examples. While every co‐crystal represents a positive example of protein–glycan interaction, structural information alone does not provide data as to whether a lectin will conclusively not bind a certain glycan. The array data, which we used here, contains an abundance of true negative examples, of lectins not binding to certain glycans, and is exclusively composed of quantitative binding data. In contrast to treating lectins as essentially black boxes,^[^ ^21^ ^]^ our sequence‐based approach further allows us to extract information from amino acid sequences and extrapolate to new lectins. This middle ground approach allowed us to combine the best of both worlds and construct a highly generalizable model that enables in‐depth analyses of lectin binding behavior. We therefore envision that LectinOracle will be a versatile platform to advance glycobiology as well as the many other life science disciplines in which lectins exercise an important role.

## 4 Experimental Section

### Data Set Construction

For the lectin–glycan data set, data from 3228 glycan arrays were manually curated from the Consortium for Functional Glycomics database, using a custom script to extract the data from the Excel files. A hundred glycan arrays were also added from the Carbohydrate Microarray Facility of Imperial College London to this data set. For all glycan arrays, glycan descriptions were converted to IUPAC‐condensed via a mapping table (Table S4, Supporting Information). Wherever possible, meta‐data was also collected about the sample, such as protein sequence, database identifiers, and expression system (Table S2, Supporting Information).

### Data Processing

First, columns that were mapped to the same glycan sequence in IUPAC‐condensed nomenclature were averaged. Then, the subset of data was selected from array experiments where the protein sequences were available to us. This resulted in a final set of 2709 glycan array experiments for training LectinOracle. All array experiments were then normalized by Z‐score transformation. Then, data from experiments were averaged using the same protein sequence in different concentrations or under different buffer conditions. Data from glycans attached to the array via different linker sequences were also averaged. These procedures were carried out to enable an interaction prediction of a protein sequence to a glycan without special consideration to environmental conditions, enabling generalizability. This resulted in 564,647 unique protein–glycan interactions which were used to train and evaluate LectinOracle. Mathematically, Z‐score transformation also resulted in negative values, which would correspond to binding below the assumed background. While this may not be relevant information, it was still chosen to supply the model with all available information to facilitate learning all relevant relationships and associations. For the purpose of interpretation, negative Z‐scores can be viewed as “non‐binding.”

### Model Training

For model training, the data set was split into a training (90%) and a test set (10%), ensuring that no proteins were present in both the training and the test set. Then, the data in both sets were converted into the format (protein sequence, glycan sequence, binding Z‐score). For the protein sequence, the 1280‐dimensional representation was retrieved from the trained ESM‐1b model. For the glycan sequence, the Python package glycowork^[^ ^19^ ^]^ was used to convert the IUPAC‐condensed sequence into a graph object, as described previously.^[^ ^8^ ^]^

LectinOracle comprised a fully connected neural network that used the ESM‐1b representations as input and a SweetNet‐based graph convolutional neural network with node embeddings to analyze glycan graphs. Results from these two arms were concatenated and used in another fully connected part, which resulted in a multisample dropout scheme^[^ ^75^ ^]^ prior to the binding prediction. Immediately before the final output, a sigmoid layer was used to scale the output to range between the maximum and minimum Z‐scores observed in the overall data set. Fully connected parts of LectinOracle constituted linear layers interspersed with leaky ReLUs, dropout layers, and batch normalization layers. All linear layers were initialized via Xavier initialization.^[^ ^76^ ^]^

All models were trained with PyTorch 1.8^[^ ^77^ ^]^ and PyTorch Geometric 1.8,^[^ ^78^ ^]^ using a single NVIDIA Tesla P100 GPU. Batch sizes of 128 were used for both training and test sets. Final hyperparameters after optimizing via cross‐validation were a starting learning rate of 0.0005, using ADAM as an optimizer, that was decayed over 80 epochs according to a cosine function. LectinOracle was trained for 100 epochs, with an early stopping criterion of 20 epochs without further improvement. For training, a mean squared error loss function was used.

### Obtaining Learned Glycan and Protein Representations

To visualize protein similarities, either ESM‐1b representations or fine‐tuned representations after training LectinOracle were used. For ESM‐1b representations, protein sequences were truncated to a maximum of 1000 amino acids, as ESM‐1b does not support substantially longer submissions. Then, this was used as an input to the trained ESM‐1b model, and the learned 1280‐dimensional representation was extracted from the final layer. For fine‐tuned protein representations, this ESM‐1b representation, together with an arbitrary dummy glycan sequence, was used as an input to LectinOracle, and the 128‐dimensional protein representation immediately prior to concatenating protein and glycan representations was extracted (i.e., after the fully connected module that fine‐tunes the ESM‐1b representation to the task of predicting protein–glycan binding).

For obtaining glycan representations, glycan sequences together with an arbitrary dummy protein ESM‐1b representation were analogously used as inputs to LectinOracle. Then, the 256‐dimensional glycan representation immediately prior to concatenating protein and glycan representations was extracted (i.e., after the pooling operations of the graph convolutional neural network). To visualize glycan clusters for Figure 1C, these glycan representations were then used to construct a cosine distance matrix. Subsequently, neighbor joining was applied to this matrix to obtain a dendrogram based on agglomerative clustering.

### Identifying Lectin Binding Specificity

To determine the binding specificity of a lectin, a library of disaccharides, trisaccharides, and *n*−1 motifs (motifs differing by one monosaccharide from full sequences in the data set) that were observed in the glycan set in this study was constructed. This choice of motifs was motivated by a compromise between interpretability and computational efficiency. Then, using a trained LectinOracle model, the ESM‐1b representation for the lectin was retrieved, and this was used as input together with all motifs to receive predicted binding scores for all motifs. Then, the background prediction value was subtracted to arrive at the final predicted binding score. For the background prediction correction, predicted binding scores were calculated for all lectins with all glycan motifs. Then, the median prediction for each motif across all lectins was calculated, and this was considered the prediction background (Table S5, Supporting Information), with the assumption that no motif should be bound by all or the majority of lectins in the data set used in this study.

### Statistical Analysis

Continuous data were depicted as mean ± s.e.m. For cases in which ranking was important, for instance, ranking of predicted motifs, Wilcoxon signed‐ranked tests were used to test for significance. Comparing two groups was done via two‐tailed Welch's *t*‐tests. For testing the equality of two variances, an F‐test was used. Linear regressions were assessed by Pearson's correlation coefficient. In all cases, significance was defined as *p* ≤ 0.05. All multiple testing was corrected with a Holm–Šídák correction. All statistical testing was done in Python 3.8 using the statsmodels package (v0.13.0) and the scipy package (v1.7.1).

## Conflict of Interest

The authors declare no conflict of interest.

## Author Contributions

D.B. conceived the method. D.B. and J.L. performed the experiments. D.B., J.L., and E.K. prepared the data set. All authors wrote and edited the manuscript.

## Supporting information

Supporting Information

Supporting Information

## Data Availability

All code used in this study can be found at https://github.com/BojarLab/LectinOracle and all data that we used is available in the supplementary tables.

## References

[1] N. Sharon , Glycobiology 2004, 14, 53R. 10.1093/glycob/cwh122 15229195

[2] M. E. Taylor , K. Drickamer , R. L. Schnaar , M. E. Etzler , A. Varki , in Essentials of Glycobiology (Eds: A. Varki , R. D. Cummings , J. D. Esko , P. Stanley , G. W. Hart , M. Aebi , A. G. Darvill , T. Kinoshita , N. H. Packer , J. H. Prestegard , R. L. Schnaar , P. H. Seeberger ), Cold Spring Harbor Laboratory Press, Cold Spring Harbor, NY 2015.

[3] K. Drickamer , M. E. Taylor , Curr. Opin. Struct. Biol. 2015, 34, 26. 26163333 10.1016/j.sbi.2015.06.003 PMC4681411

[4] T. O. Edinger , M. O. Pohl , S. Stertz , J. Gen. Virol. 2014, 95, 263. 24225499 10.1099/vir.0.059477-0

[5] T. K. Dam , T. A. Gerken , C. F. Brewer , Biochemistry 2009, 48, 3822. 19292456 10.1021/bi9002919 PMC2691598

[6] M. E. Taylor , K. Drickamer , Curr. Opin. Struct. Biol. 2014, 28, 14. 25102772 10.1016/j.sbi.2014.07.003 PMC4444583

[7] A. Varki , Glycobiology 2017, 27, 3. 27558841 10.1093/glycob/cww086 PMC5884436

[8] R. Burkholz , J. Quackenbush , D. Bojar , Cell Rep. 2021, 35, 109251. 34133929 10.1016/j.celrep.2021.109251 PMC9208909

[9] O. Shimomura , T. Oda , H. Tateno , Y. Ozawa , S. Kimura , S. Sakashita , M. Noguchi , J. Hirabayashi , M. Asashima , N. Ohkohchi , Mol. Cancer Ther. 2018, 17, 183. 28939555 10.1158/1535-7163.MCT-17-0232

[10] S. Žurga , M. P. Nanut , J. Kos , J. Sabotič , Oncotarget 2017, 8, 26896. 28460472 10.18632/oncotarget.15849 PMC5432305

[11] F. Bonnardel , J. Mariethoz , S. Pérez , A. Imberty , F. Lisacek , Nucleic Acids Res. 2021, 49, D1548. 33174598 10.1093/nar/gkaa1019 PMC7778903

[12] K. He , X. Zhang , S. Ren , J. Sun , in 2015 IEEE Int. Conf. Computer Vision (ICCV), IEEE, Santiago, Chile 2015, pp. 1026–1034.

[13] H. Li , Natl. Sci. Rev. 2018, 5, 24.

[14] J. Jumper , R. Evans , A. Pritzel , T. Green , M. Figurnov , O. Ronneberger , K. Tunyasuvunakool , R. Bates , A. Žídek , A. Potapenko , A. Bridgland , C. Meyer , S. A. A. Kohl , A. J. Ballard , A. Cowie , B. Romera‐Paredes , S. Nikolov , R. Jain , J. Adler , T. Back , S. Petersen , D. Reiman , E. Clancy , M. Zielinski , M. Steinegger , M. Pacholska , T. Berghammer , S. Bodenstein , D. Silver , O. Vinyals , A. W. Senior , K. Kavukcuoglu , P. Kohli , D. Hassabis , Nature 2021, 596, 583. 34265844 10.1038/s41586-021-03819-2 PMC8371605

[15] M. Baek , F. DiMaio , I. Anishchenko , J. Dauparas , S. Ovchinnikov , G. R. Lee , J. Wang , Q. Cong , L. N. Kinch , R. D. Schaeffer , C. Millán , H. Park , C. Adams , C. R. Glassman , A. DeGiovanni , J. H. Pereira , A. V. Rodrigues , A. A. van Dijk , A. C. Ebrecht , D. J. Opperman , T. Sagmeister , C. Buhlheller , T. Pavkov‐Keller , M. K. Rathinaswamy , U. Dalwadi , C. K. Yip , J. E. Burke , K. C. Garcia , N. V. Grishin , P. D. Adams , R. J. Read , D. Baker , Science 2021, eabj8754.

[16] A. Rives , J. Meier , T. Sercu , S. Goyal , Z. Lin , J. Liu , D. Guo , M. Ott , C. L. Zitnick , J. Ma , R. Fergus , Proc. Natl. Acad. Sci. USA 2021, 118, e2016239118. 33876751 10.1073/pnas.2016239118 PMC8053943

[17] D. Bojar , R. K. Powers , D. M. Camacho , J. J. Collins , Cell Host Microbe 2021, 29, 132.e3. 33120114 10.1016/j.chom.2020.10.004

[18] L. Thomès , D. Bojar , Front. Mol. Biosci. 2021, 8, 755577. 34631801 10.3389/fmolb.2021.755577 PMC8492980

[19] L. Thomès , R. Burkholz , D. Bojar , Glycobiology 2021, cwab067. 10.1093/glycob/cwab067 PMC8600276 34192308

[20] L. Coff , J. Chan , P. A. Ramsland , A. J. Guy , BMC Bioinf. 2020, 21, 42. 10.1186/s12859-020-3374-4 PMC7001330 32019496

[21] E. J. Carpenter , S. Seth , N. Yue , R. Greiner , R. Derda , bioRxiv 2021. https://www.biorxiv.org/content/10.1101/2021.05.28.446094v1

[22] Y. Cao , Y. Shen , Bioinformatics 2021, btab198.33755048

[23] I. Letunic , P. Bork , Nucleic Acids Res. 2021, 49, W293. 33885785 10.1093/nar/gkab301 PMC8265157

[24] D. Kletter , S. Singh , M. Bern , B. B. Haab , Mol. Cell. Proteomics 2013, 12, 1026. 23399549 10.1074/mcp.M112.026641 PMC3617327

[25] N. Shibuya , I. J. Goldstein , W. F. Broekaert , M. Nsimba‐Lubaki , B. Peeters , W. J. Peumans , J. Biol. Chem. 1987, 262, 1596. 3805045

[26] F. Fukumori , N. Takeuchi , T. Hagiwara , H. Ohbayashi , T. Endo , N. Kochibe , Y. Nagata , A. Kobata , J. Biochem. 1990, 107, 190. 2193930 10.1093/oxfordjournals.jbchem.a123024

[27] V. S. R. Rao , K. Lam , P. K. Qasba , J. Biomol. Struct. Dyn. 1998, 15, 853. 9619508 10.1080/07391102.1998.10508207

[28] J.‐F. Sanchez , J. Lescar , V. Chazalet , A. Audfray , J. Gagnon , R. Alvarez , C. Breton , A. Imberty , E. P. Mitchell , J. Biol. Chem. 2006, 281, 20171. 16704980 10.1074/jbc.M603452200

[29] K. A. Maupin , D. Liden , B. B. Haab , Glycobiology 2012, 22, 160. 21875884 10.1093/glycob/cwr128 PMC3230281

[30] B. M. Cummins , J. T. Garza , G. L. Coté , Anal. Chem. 2013, 85, 5397. 23627407 10.1021/ac303689j PMC3753689

[31] C. J. Day , L. E. Hartley‐Tassell , K. L. Seib , J. Tiralongo , N. Bovin , S. Savino , V. Masignani , M. P. Jennings , Biochem. Biophys. Res. Commun. 2019, 513, 287. 30954224 10.1016/j.bbrc.2019.03.092

[32] N. Huwa , O. H. Weiergräber , C. Kirsch , U. Schaffrath , T. Classen , IJMS 2021, 22, 5639. 34073266 10.3390/ijms22115639 PMC8197871

[33] Y. Bourne , C. H. Astoul , V. Zamboni , W. J. Peumans , L. Menu‐Bouaouiche , E. J. M. Van Damme , A. Barre , P. Rougé , Biochem. J. 2002, 364, 173. 11988090 10.1042/bj3640173 PMC1222559

[34] K. D. Weynberg , M. J. Allen , K. Ashelford , D. J. Scanlan , W. H. Wilson , Environ. Microbiol. 2009, 11, 2821. 19650882 10.1111/j.1462-2920.2009.01991.x

[35] A. B. Boraston , D. Wang , R. D. Burke , J. Biol. Chem. 2006, 281, 35263. 16987809 10.1074/jbc.M607620200

[36] M. Perduca , M. Bovi , L. Destefanis , D. Nadali , L. Fin , F. Parolini , D. Sorio , M. E. Carrizo , H. L. Monaco , Glycobiology 2021, cwab059. 10.1093/glycob/cwab059 34192315

[37] J. Tiralongo , O. Cooper , T. Litfin , Y. Yang , R. King , J. Zhan , H. Zhao , N. Bovin , C. J. Day , Y. Zhou , Sci. Rep. 2018, 8, 13139. 30177739 10.1038/s41598-018-31241-8 PMC6120924

[38] D. Bojar , L. Meche , G. Meng , W. Eng , D. F. Smith , R. D. Cummings , L. K. Mahal , bioRxiv 2021. https://www.biorxiv.org/content/10.1101/2021.08.31.458439v2 10.1021/acschembio.1c00689 PMC9679999 35084820

[39] R. D. Cummings , F.‐T. Liu , G. R. Vasta , in Essentials of Glycobiology (Eds: A. Varki , R. D. Cummings , J. D. Esko , P. Stanley , G. W. Hart , M. Aebi , A. G. Darvill , T. Kinoshita , N. H. Packer , J. H. Prestegard , R. L. Schnaar , P. H. Seeberger ), Cold Spring Harbor Laboratory Press, Cold Spring Harbor, NY 2015. 27010055

[40] M. Tsaneva , E. J. M. Van Damme , Glycoconjugate J. 2020, 37, 533. 10.1007/s10719-020-09942-y PMC7455784 32860551

[41] W. J. Peumans , J. M. van Damme , A. Barre , P. Rougé , in The Molecular Immunology of Complex Carbohydrates 2 (Ed: A. M. Wu ), Springer US, Boston, MA 2001, pp. 27–54.

[42] Y.‐F. Wang , C.‐F. Chang , H.‐P. Tsai , C.‐Y. Chi , I.‐J. Su , J.‐R. Wang , PLoS One 2018, 13, e0196727. 29746492 10.1371/journal.pone.0196727 PMC5945028

[43] F. Bonnardel , J. Mariethoz , S. Salentin , X. Robin , M. Schroeder , S. Perez , F. Lisacek , A. Imberty , Nucleic Acids Res. 2019, 47, D1236. 30239928 10.1093/nar/gky832 PMC6323968

[44] S. Yao , R. You , S. Wang , Y. Xiong , X. Huang , S. Zhu , Nucleic Acids Res. 2021, 49, W469. 34038555 10.1093/nar/gkab398 PMC8262706

[45] L. van der Maaten , G. Hinton , J. Mach. Learn. Res. 2008, 9, 2579.

[46] J. H. Ward , J. Am. Stat. Assoc. 1963, 58, 236.

[47] L. Byrd‐Leotis , R. D. Cummings , D. A. Steinhauer , IJMS 2017, 18, 1541. 10.3390/ijms18071541 PMC5536029 28714909

[48] B. Timmermans , A. De Las Peñas , I. Castaño , P. Van Dijck , JoF 2018, 4, 60. 10.3390/jof4020060 PMC6023314 29783771

[49] P. Stanley , R. D. Cummings , in Essentials of Glycobiology (Eds: A. Varki , R. D. Cummings , J. D. Esko , P. Stanley , G. W. Hart , M. Aebi , A. G. Darvill , T. Kinoshita , N. H. Packer , J. H. Prestegard , R. L. Schnaar , P. H. Seeberger ), Cold Spring Harbor Laboratory Press, Cold Spring Harbor, NY 2015. 27010055

[50] E. Byres , A. W. Paton , J. C. Paton , J. C. Löfling , D. F. Smith , M. C. J. Wilce , U. M. Talbot , D. C. Chong , H. Yu , S. Huang , X. Chen , N. M. Varki , A. Varki , J. Rossjohn , T. Beddoe , Nature 2008, 456, 648. 18971931 10.1038/nature07428 PMC2723748

[51] M. A. Pak , K. A. Markhieva , M. S. Novikova , D. S. Petrov , I. S. Vorobyev , E. S. Maksimova , F. A. Kondrashov , D. N. Ivankov , bioRxiv 2021. https://www.biorxiv.org/content/10.1101/2021.09.19.460937v1.abstract 10.1371/journal.pone.0282689 PMC10019719 36928239

[52] D. Hu , H. Tateno , A. Kuno , R. Yabe , J. Hirabayashi , J. Biol. Chem. 2012, 287, 20313. 22493425 10.1074/jbc.M112.351965 PMC3370213

[53] C. Gao , K. Stavenhagen , B. Eckmair , T. R. McKitrick , A. Y. Mehta , Y. Matsumoto , A. M. McQuillan , M. S. Hanes , D. Eris , K. J. Baker , N. Jia , M. Wei , J. Heimburg‐Molinaro , B. Ernst , R. D. Cummings , Sci. Adv. 2021, 7, eabf6834. 34108208 10.1126/sciadv.abf6834 PMC8189592

[54] S. Wang , C. Chen , M. R. Gadi , V. Saikam , D. Liu , H. Zhu , R. Bollag , K. Liu , X. Chen , F. Wang , P. G. Wang , P. Ling , W. Guan , L. Li , Nat. Commun. 2021, 12, 3573. 34117223 10.1038/s41467-021-23428-x PMC8196059

[55] A. Geissner , A. Reinhardt , C. Rademacher , T. Johannssen , J. Monteiro , B. Lepenies , M. Thépaut , F. Fieschi , J. Mrázková , M. Wimmerova , F. Schuhmacher , S. Götze , D. Grünstein , X. Guo , H. S. Hahm , J. Kandasamy , D. Leonori , C. E. Martin , S. G. Parameswarappa , S. Pasari , M. K. Schlegel , H. Tanaka , G. Xiao , Y. Yang , C. L. Pereira , C. Anish , P. H. Seeberger , Proc. Natl. Acad. Sci. USA 2019, 116, 1958. 30670663 10.1073/pnas.1800853116 PMC6369816

[56] X. Song , H. Yu , X. Chen , Y. Lasanajak , M. M. Tappert , G. M. Air , V. K. Tiwari , H. Cao , H. A. Chokhawala , H. Zheng , R. D. Cummings , D. F. Smith , J. Biol. Chem. 2011, 286, 31610. 21757734 10.1074/jbc.M111.274217 PMC3173124

[57] M. A. Naarding , I. S. Ludwig , F. Groot , B. Berkhout , T. B. H. Geijtenbeek , G. Pollakis , W. A. Paxton , J. Clin. Invest. 2005, 115, 3256. 16239964 10.1172/JCI25105 PMC1257537

[58] A. Mishra , A. Behura , S. Mawatwal , A. Kumar , L. Naik , S. S. Mohanty , D. Manna , P. Dokania , A. Mishra , S. K. Patra , R. Dhiman , Food Chem. Toxicol. 2019, 134, 110827. 31542433 10.1016/j.fct.2019.110827 PMC7115788

[59] G. R. Vasta , H. Ahmed , S. Tasumi , E. W. Odom , K. Saito , in Current Topics in Innate Immunity (Ed: J. D. Lambris ), Springer New York, New York, NY 2007, pp. 389–406. 10.1007/978-0-387-71767-8_27 17892226

[60] T. Ichimiya , S. Nishihara , S. Takase‐Yoden , H. Kida , K. Aoki‐Kinoshita , Bioinformatics 2014, 30, 706. 24135264 10.1093/bioinformatics/btt573

[61] L. Byrd‐Leotis , N. Jia , S. Dutta , J. F. Trost , C. Gao , S. F. Cummings , T. Braulke , S. Müller‐Loennies , J. Heimburg‐Molinaro , D. A. Steinhauer , R. D. Cummings , Sci. Adv. 2019, 5, eaav2554. 30788437 10.1126/sciadv.aav2554 PMC6374103

[62] C.‐M. Liao , S.‐Y. Chang , S.‐C. Chen , C.‐P. Chio , Int. J. Infect. Dis. 2009, 13, 589. 19111494 10.1016/j.ijid.2008.09.019

[63] F. Bonnardel , S. M. Haslam , A. Dell , T. Feizi , Y. Liu , V. Tajadura‐Ortega , Y. Akune , L. Sykes , P. R. Bennett , D. A. MacIntyre , F. Lisacek , A. Imberty , npj Biofilms Microbiomes 2021, 7, 49. 34131152 10.1038/s41522-021-00220-9 PMC8206207

[64] J. Pizarro‐Cerdá , P. Cossart , Cell 2006, 124, 715. 16497583 10.1016/j.cell.2006.02.012

[65] R. Matos , I. Amorim , A. Magalhães , F. Haesebrouck , F. Gärtner , C. A. Reis , Front. Mol. Biosci. 2021, 8, 656439. 34026832 10.3389/fmolb.2021.656439 PMC8138122

[66] S. Dedola , M. D. Rugen , R. J. Young , R. A. Field , ChemBioChem 2020, 21, 423. 31317590 10.1002/cbic.201900377 PMC7463168

[67] M. L. Bulyk , X. Huang , Y. Choo , G. M. Church , Proc. Natl. Acad. Sci. USA 2001, 98, 7158. 11404456 10.1073/pnas.111163698 PMC34639

[68] L. Liu , P. Chopra , X. Li , M. A. Wolfert , S. M. Tompkins , G.‐J. Boons , Biochemistry 2021, 7, 1009. 10.1021/acscentsci.1c00010 PMC8227597 34235261

[69] X. Zou , M. Yoshida , C. Nagai‐Okatani , J. Iwaki , A. Matsuda , B. Tan , K. Hagiwara , T. Sato , Y. Itakura , E. Noro , H. Kaji , M. Toyoda , Y. Zhang , H. Narimatsu , A. Kuno , Sci. Rep. 2017, 7, 43560. 28262709 10.1038/srep43560 PMC5337905

[70] M. Sojitra , S. Sarkar , J. Maghera , E. Rodrigues , E. J. Carpenter , S. Seth , D. Ferrer Vinals , N. J. Bennett , R. Reddy , A. Khalil , X. Xue , M. R. Bell , R. B. Zheng , P. Zhang , C. Nycholat , J. J. Bailey , C.‐C. Ling , T. L. Lowary , J. C. Paulson , M. S. Macauley , R. Derda , Nat. Chem. Biol. 2021, 17, 806. 33958792 10.1038/s41589-021-00788-5 PMC8380037

[71] O. C. Grant , H. M. Smith , D. Firsova , E. Fadda , R. J. Woods , Glycobiology 2014, 24, 17. 24056723 10.1093/glycob/cwt083 PMC3854501

[72] T. M. Lucas , C. Gupta , M. O. Altman , E. Sanchez , M. R. Naticchia , P. Gagneux , A. Singharoy , K. Godula , Chem 2021. 10.1016/j.chempr.2021.09.015 PMC8726012 34993358

[73] J. S. Temme , C. T. Campbell , J. C. Gildersleeve , Faraday Discuss. 2019, 219, 90. 31338503 10.1039/c9fd00021f PMC9335900

[74] H. Tateno , S. Nakamura‐Tsuruta , J. Hirabayashi , Nat. Protoc. 2007, 2, 2529. 17947995 10.1038/nprot.2007.357

[75] H. Inoue , arXiv:1905.09788 [cs, stat] 2019.

[76] X. Glorot , Y. Bengio , in *Proc. Thirteenth Int. Conf. Artificial Intelligence and Statistics, PMLR* 2010, 9, 249.

[77] A. Paszke , S. Gross , F. Massa , A. Lerer , J. Bradbury , G. Chanan , T. Killeen , Z. Lin , N. Gimelshein , L. Antiga , A. Desmaison , A. Köpf , E. Yang , Z. DeVito , M. Raison , A. Tejani , S. Chilamkurthy , B. Steiner , L. Fang , J. Bai , S. Chintala , arXiv:1912.01703 [cs, stat] 2019.

[78] M. Fey , J. E. Lenssen , arXiv:1903.02428 [cs, stat] 2019.

