# Supplementary material for: LectinOracle: A Generalizable Deep Learning Model for Lectin–Glycan Binding Prediction
Source: Adv Sci (Weinh). 2021 Dec 4;9(1):2103807. doi: 10.1002/advs.202103807 (PMC8728848; doi:10.1002/advs.202103807)
Supplement: Supplementary file 1 — Supporting Information [file ADVS-9-2103807-s002.pdf]

ADVANCED SCIENCE
Open Access

Supporting Information

for *Adv. Sci.*, DOI: 10.1002/advs.202103807

LectinOracle – A Generalizable Deep Learning Model for Lectin-Glycan Binding Prediction

Jon Lundstrøm[1], Emma Korhonen[1], Frédérique Lisacek[2], Daniel Bojar[1,]*

LectinOracle – A Generalizable Deep Learning Model for Lectin-Glycan Binding Prediction

Jon Lundstrøm[1], Emma Korhonen[1], Frédérique Lisacek[2], Daniel Bojar[1,*]

[1]Department of Chemistry and Molecular Biology, University of Gothenburg, Gothenburg, Sweden. Wallenberg Centre for Molecular and Translational Medicine, University of Gothenburg, Gothenburg, Sweden.

[2]Swiss Institute of Bioinformatics, Geneva, Switzerland. Computer Science Department, UniGe, Geneva, Switzerland. Section of Biology, UniGe, Geneva, Switzerland.

[*]Corresponding author: daniel.bojar@gu.se

Supplementary Figures

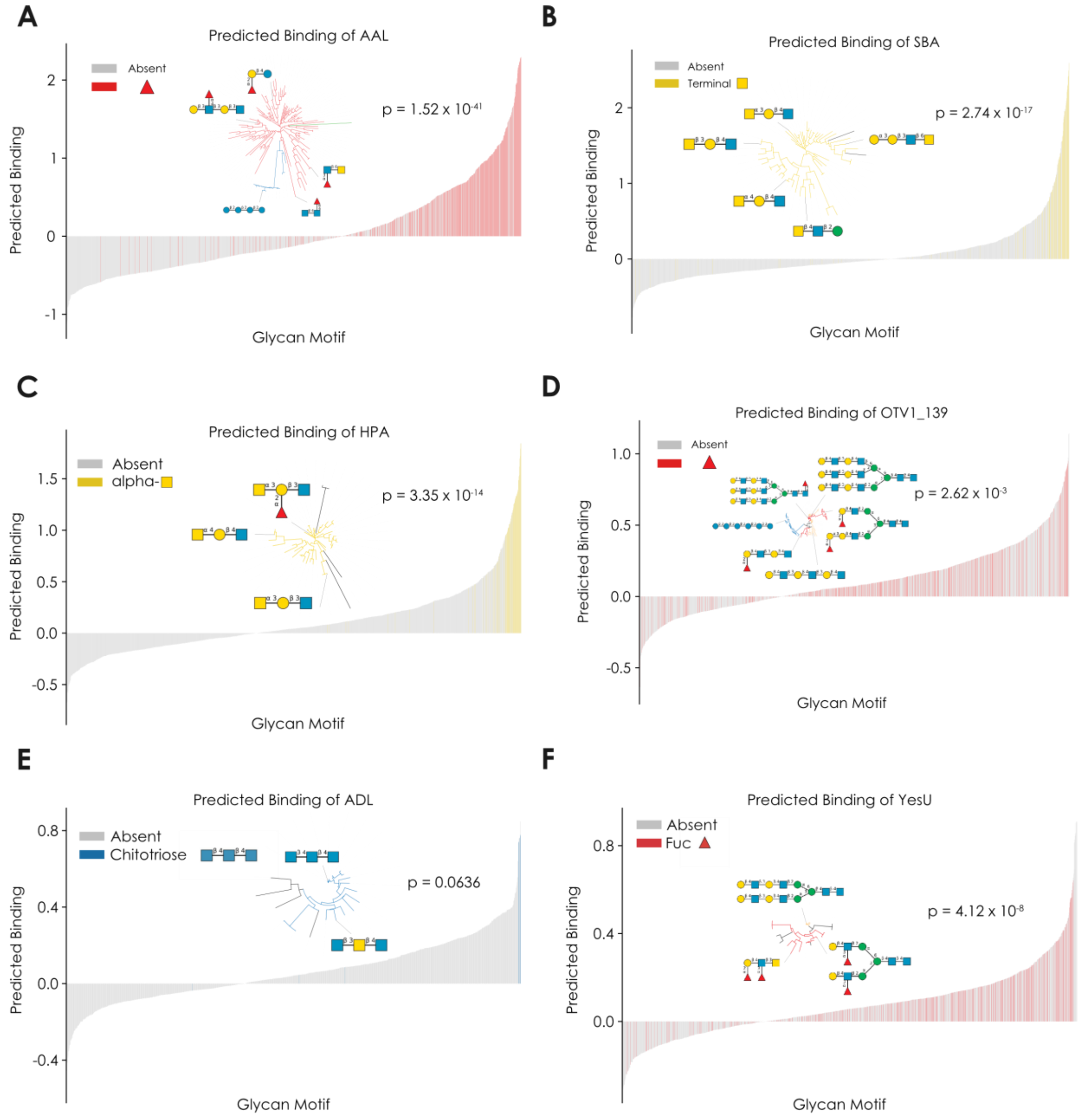

Figure S1. Glycan-binding specificity of various lectins with LectinOracle. A-F) For a range of glycan motifs, a trained LectinOracle model was used to predict their binding to the lectins AAL (A), SBA (B), HPA (C), OTV1_139 (D), ADL (E), and YesU (F) with their literature-annotated binding motifs or enriched binding motifs colored in. Motif enrichment was tested via one-sided Wilcoxon signed-rank tests, with the p-value shown in each panel. Dendrograms of bound motifs are shown similar to Fig. 1C.

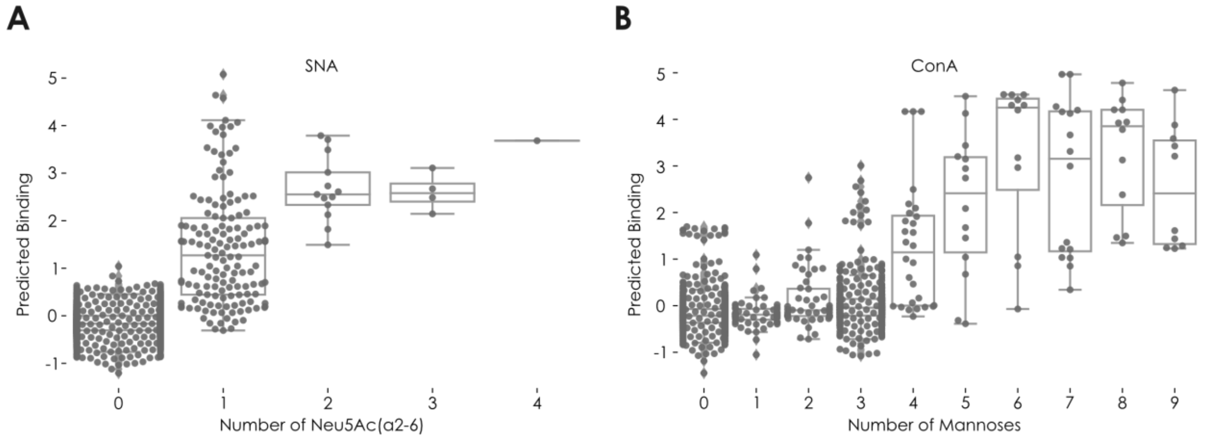

Figure S2. Correlation of binding predictions with the number of binding epitopes. A-B) For the lectins SNA (A) and ConA (B), we used a trained LectinOracle model to obtain binding predictions for a range of glycan motifs and full glycans. Then, we counted the occurrence of the literature-defined binding motif for the respective lectin in each glycan motif and plotted the predicted binding as a box plot depicting mean, quartiles, and data distribution.

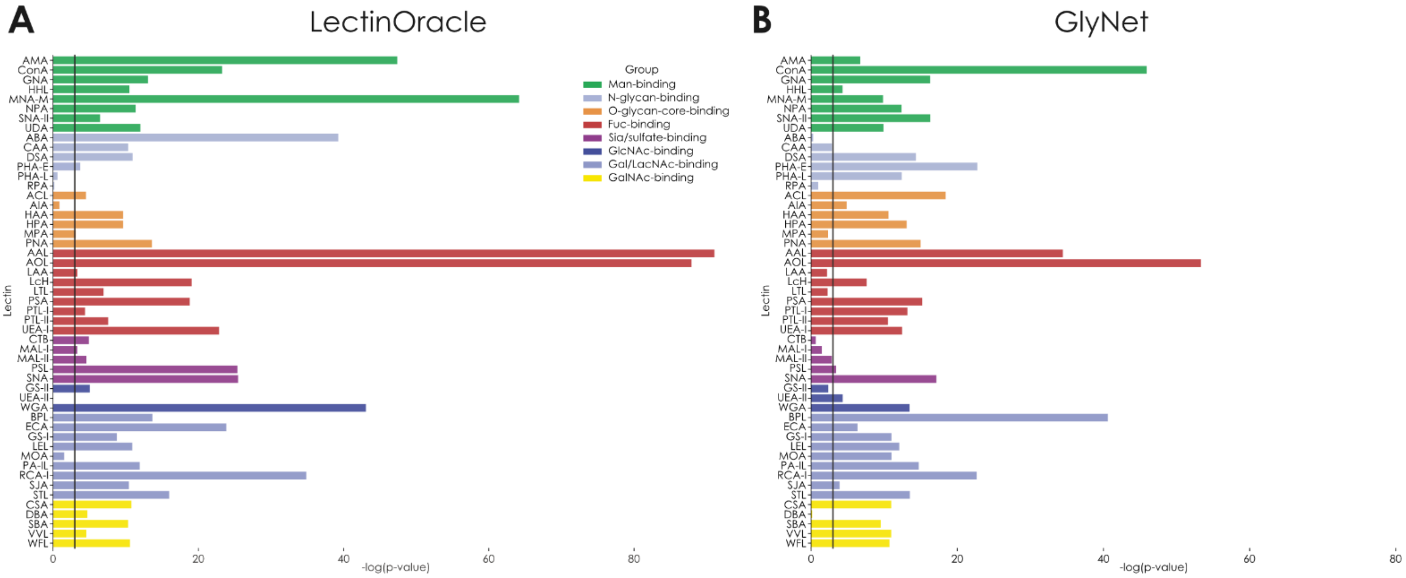

Figure S3. Validating LectinOracle predictions on 51 commonly used lectins. A-B) For 51 commonly used lectins, we used one-sided Wilcoxon signed-ranked tests to evaluate whether the predominant binding motif, resulting from a combination of machine learning and expert manual annotation, was also predicted by LectinOracle (A) or GlyNet (B). Lectins are grouped in related classes according to their binding specificity. Shown p-values are log-transformed and a vertical line indicates the minimum value required for statistical significance (equivalent to $p = 0.05$).

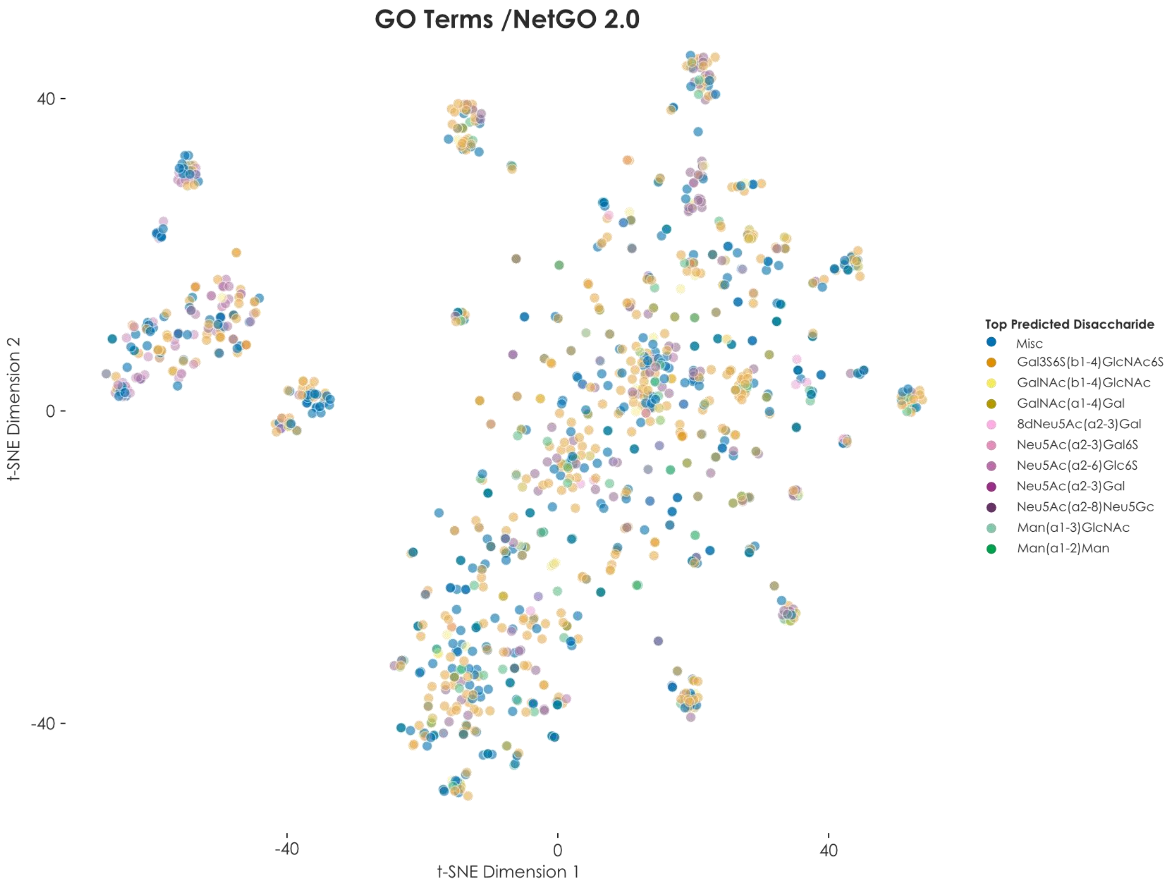

Figure S4. Clustering lectins by predicted GO term annotation. GO term annotations scores, predicted by NetGO 2.0, for all lectins used for training LectinOracle were used as features for t-SNE visualization, colored by the top predicted disaccharide, analogous to Fig. 2A-B.

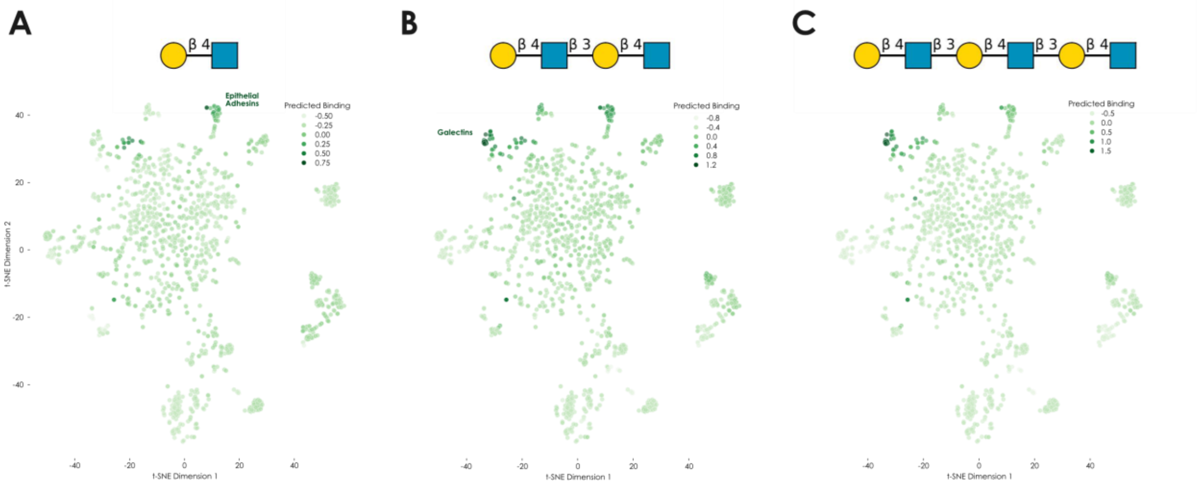

Figure S5. LectinOracle can distinguish repeats of a disaccharide motif. A-C) For the motifs type II LacNAc, di-LacNAc, and tri-LacNAc, we obtained the predicted binding from a trained LectinOracle model for all lectins and colored the learned representation from Figure 2B accordingly. Clusters of lectins exhibiting elevated predicted binding to at least one motif are annotated with enriched lectins.

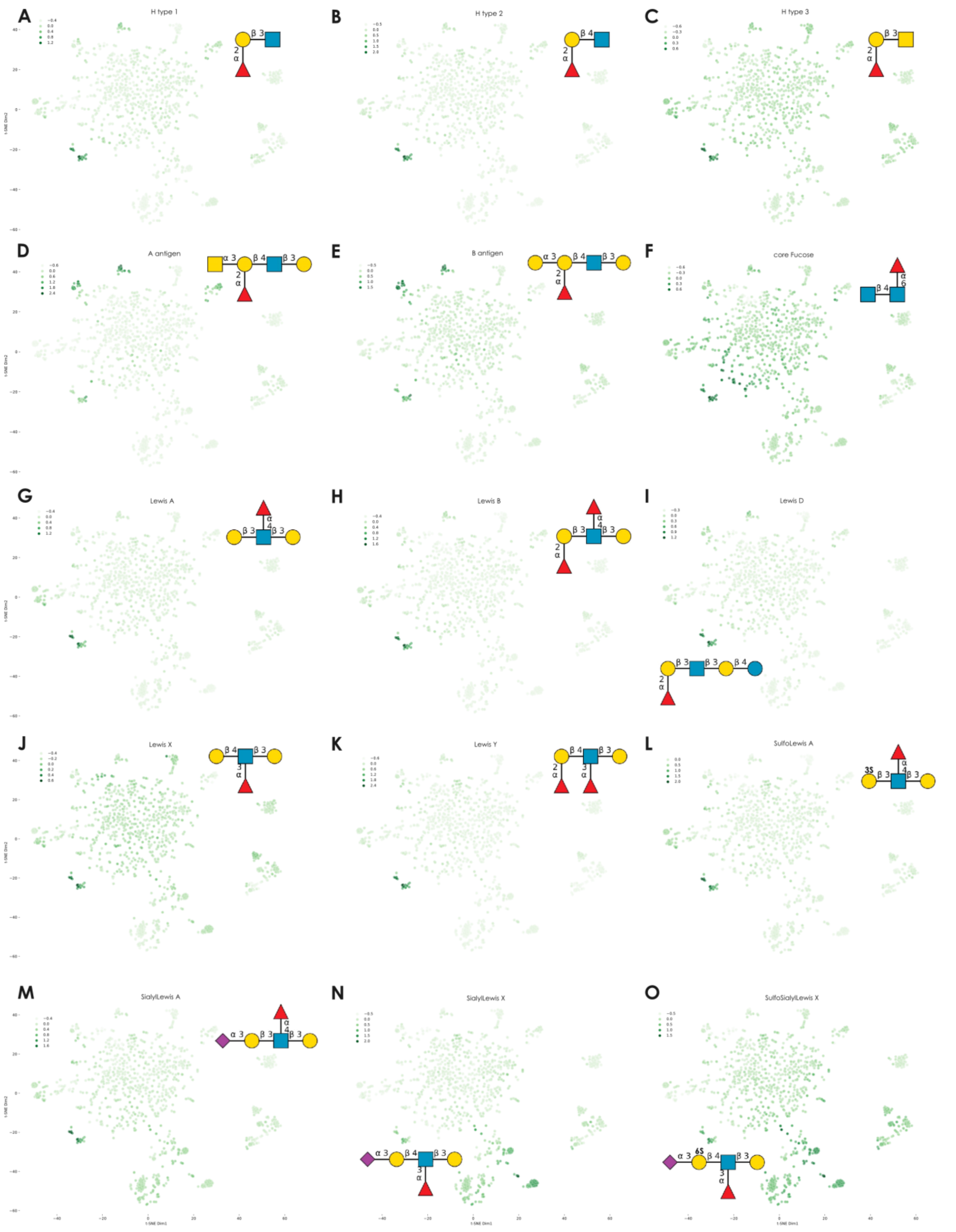

Figure S6. Predicted binding of lectins to important glycan motifs. A-O) We used a trained LectinOracle model to infer the binding of all lectins in our dataset to a range of important glycan motifs (A-O) and then colored the t-SNE visualized lectin representation (see Figure 2B) according to the predicted binding to the respective glycan motif. The glycan motifs are shown for each panel in SNFG format.

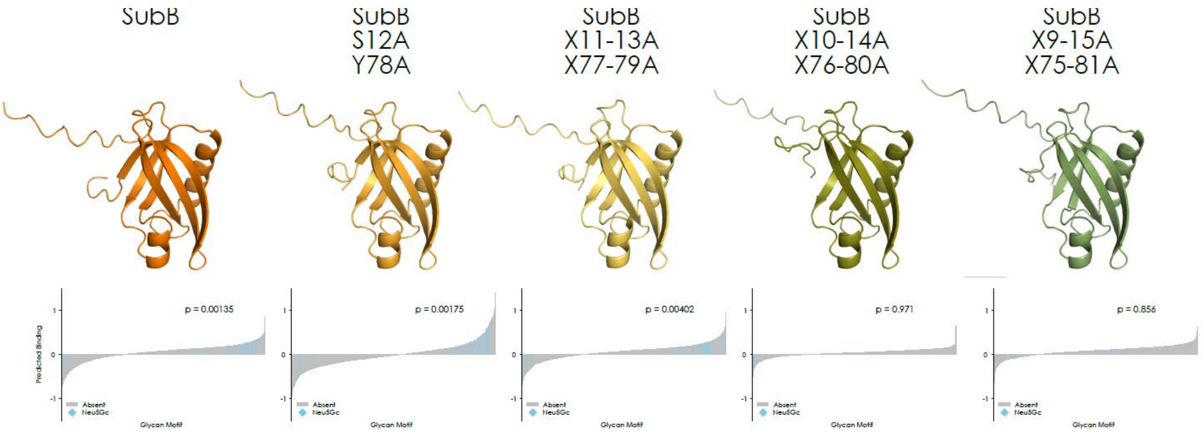

Figure S7. Sensitivity of LectinOracle predictions for SubB - Neu5Gc interactions. Similar to Figure 2C, we retrieved the binding predictions for a range of glycan motifs for SubB and various alanine-substitution mutants from a trained LectinOracle model. Binding predictions are colored by the presence or absence of Neu5Gc and the enrichment p-values were calculated via a one-sided Wilcoxon signed-rank test. We note that there are far fewer Neu5Gc-containing motifs than for Neu5Ac, due to a dearth of Neu5Gc-containing glycans on the glycan array we used to train LectinOracle. Protein structure predictions made with AlphaFold2 are shown for the wild-type protein and each mutant.

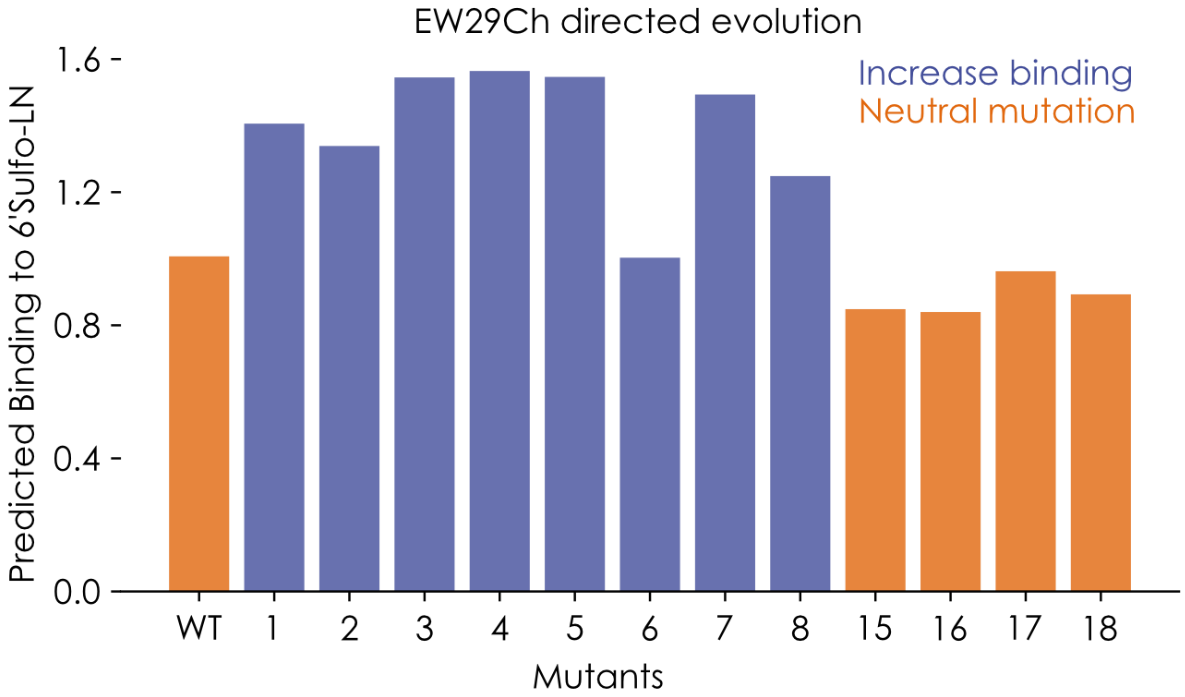

Figure S8. Using LectinOracle to predict the effect of mutations in lectin directed evolution. The binding of wildtype EW29Ch and various mutants, from a directed evolution experiment, to 6'-sulfo-LacNAc (Gal6S(β1-4)GlcNAc) was predicted using LectinOracle. Mutants that were experimentally found to increase binding to 6'-sulfo-LacNAc are colored in blue, while neutral mutants are colored in orange.

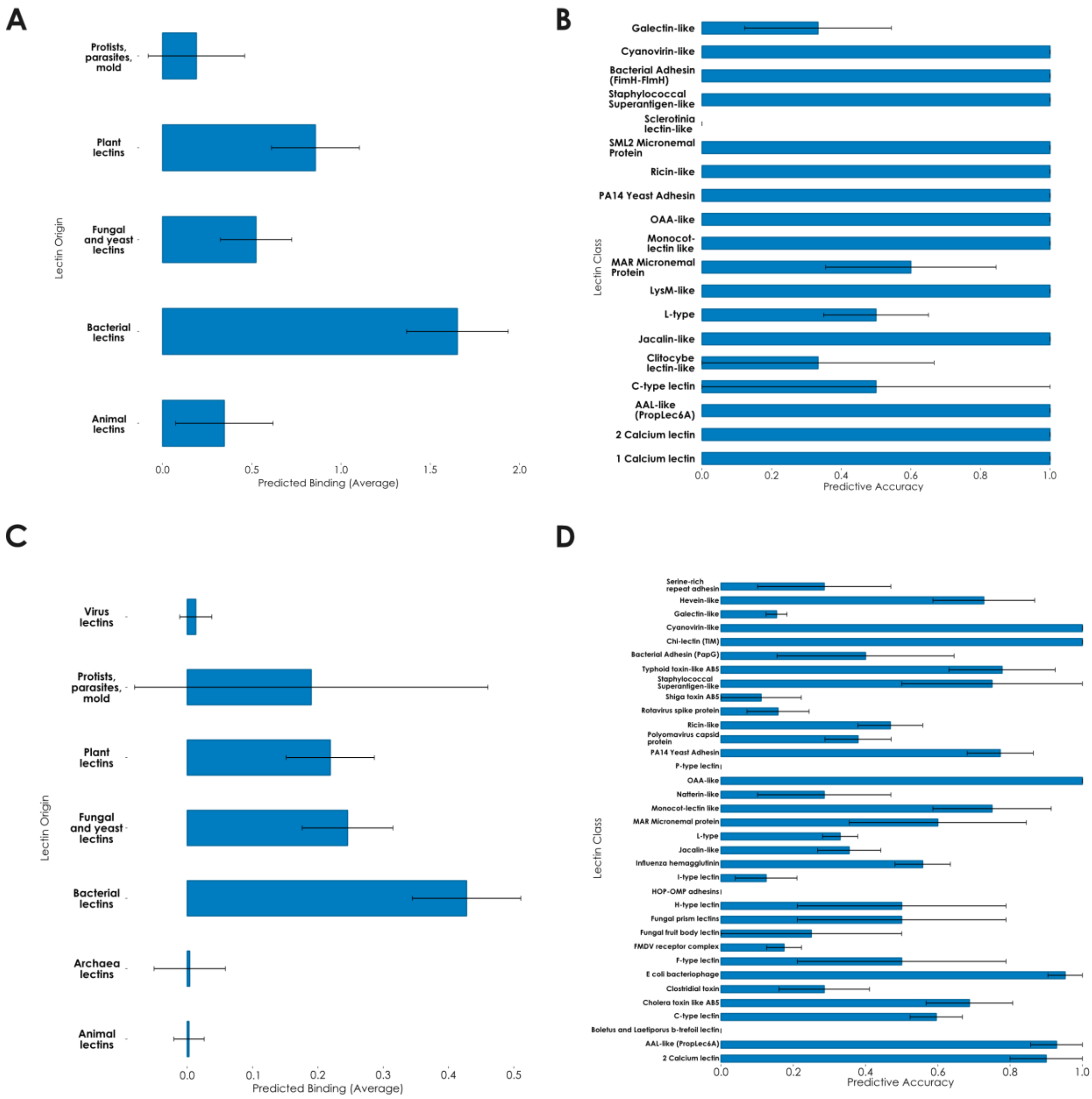

Figure S9. Comparing LectinOracle predictions and UniLectin3D crystal structures. A-B) Predictions for UniLectin3D proteins contained in our dataset. For all lectin-glycan structures for which the lectin was part of our dataset, we used a trained LectinOracle model to predict the binding of the lectin-glycan pair and show the average predicted binding grouped by lectin origin (A) or class (B). **C-D)** Predictions for all UniLectin3D proteins. For all lectin-glycan structures, we used a trained LectinOracle model to predict the binding of the lectin-glycan pair and show the average predicted binding grouped by lectin origin (C) or class (D), for classes with at least three proteins in UniLectin3D. Shown are means ± s.e.m.

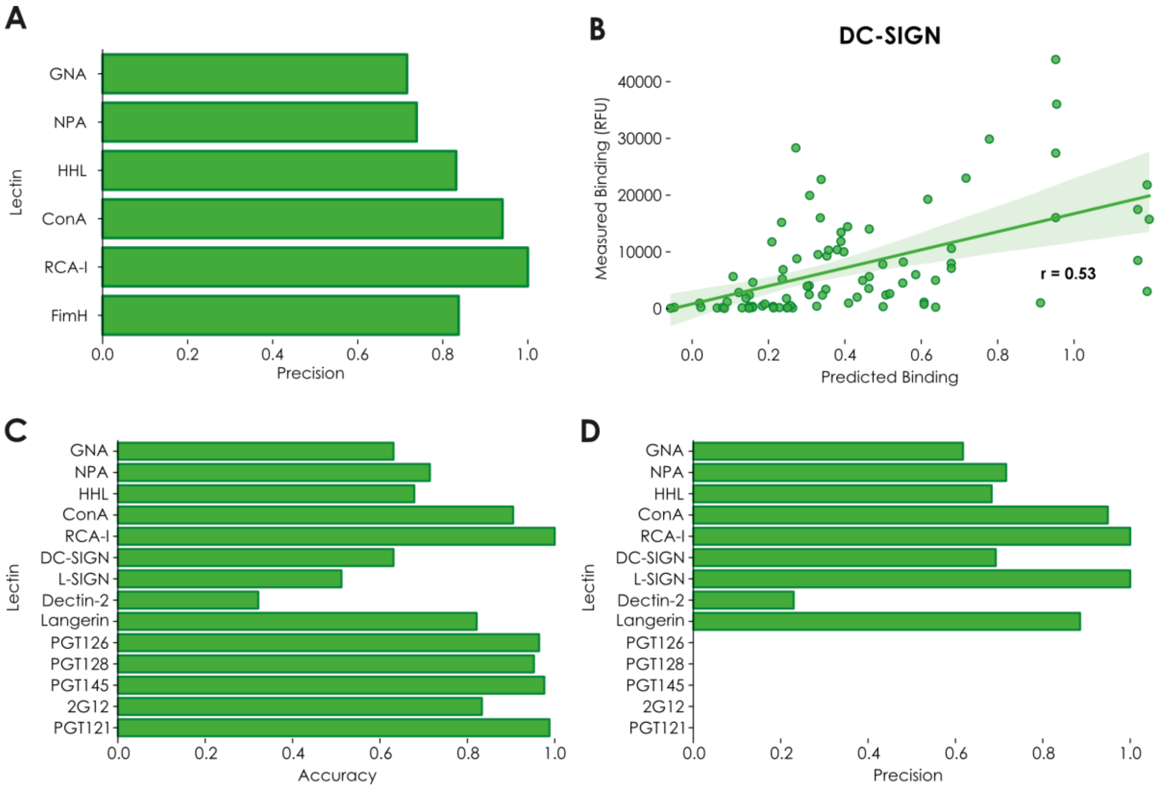

Figure S10. Additional validation of LectinOracle with data from the oligomannose array. A) Precision of LectinOracle predictions for lectins tested on the oligomannose array. For each lectin-glycan pair, we assigned it the label "bound" or "predicted bound" if the observed relative fluorescence units (RFU) were at least 10% of the maximum RFU or if the predicted binding was at least 0.5, respectively. **B)** Correlating experimentally observed binding with predictions for the lectin DC-SIGN. Correlations between experimental data and predictions were done via fitting a linear regression and r represents Pearson's correlation coefficient. **C-D)** Validating LectinOracle on another set of lectins tested on the oligomannose array. For a set of plant lectins, mammalian lectins, and antibodies, we compared LectinOracle predictions similar to (A) and depict predictive accuracy (C) and precision (D).

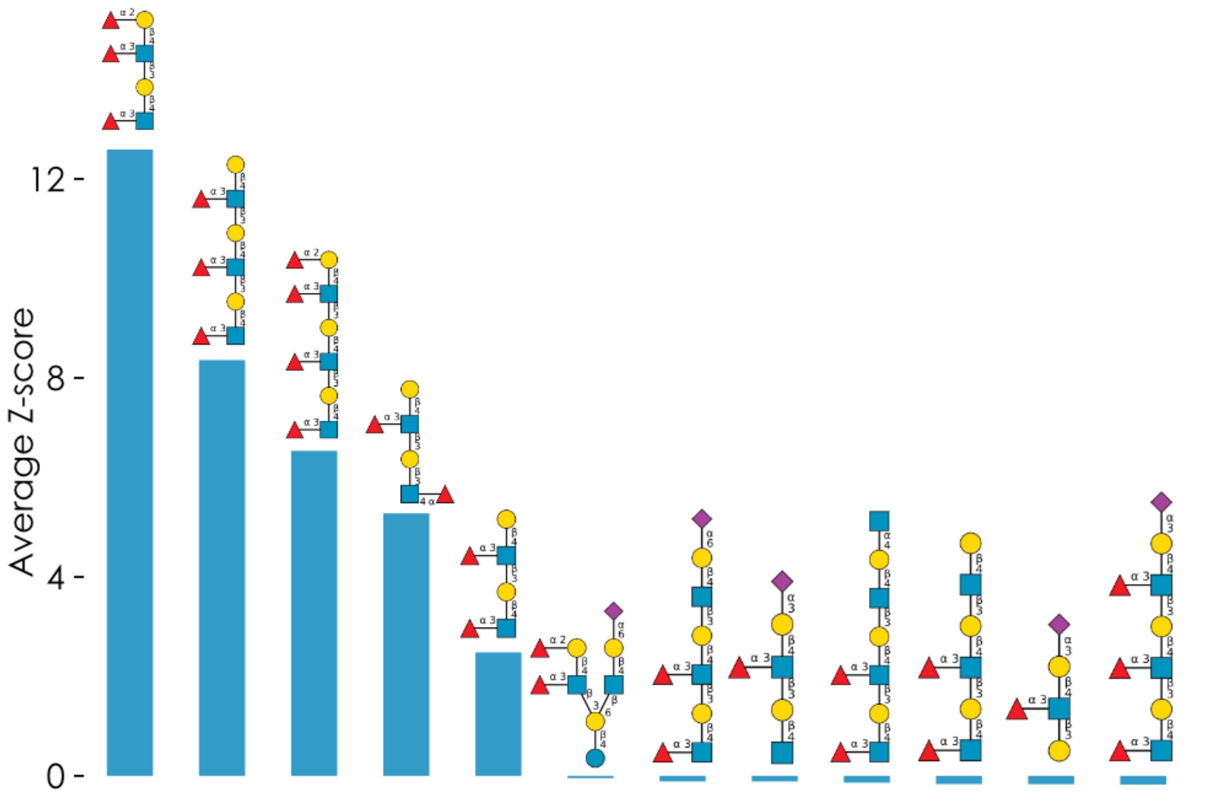

Figure S11. Elucidating the binding specificity of human DC-SIGN. We used glycowork to find all glycans across our glycan arrays that contained the LewisX motif (Fuc(α1-3)[Gal(β1-4)]GlcNAc(β1-3); LeX) and that had at least one measured interaction with human DC-SIGN. Shown are the Z-score values of these interactions, averaged across all arrays assayed with DC-SIGN, and the corresponding glycans in the symbol nomenclature for glycans (SNFG) format.

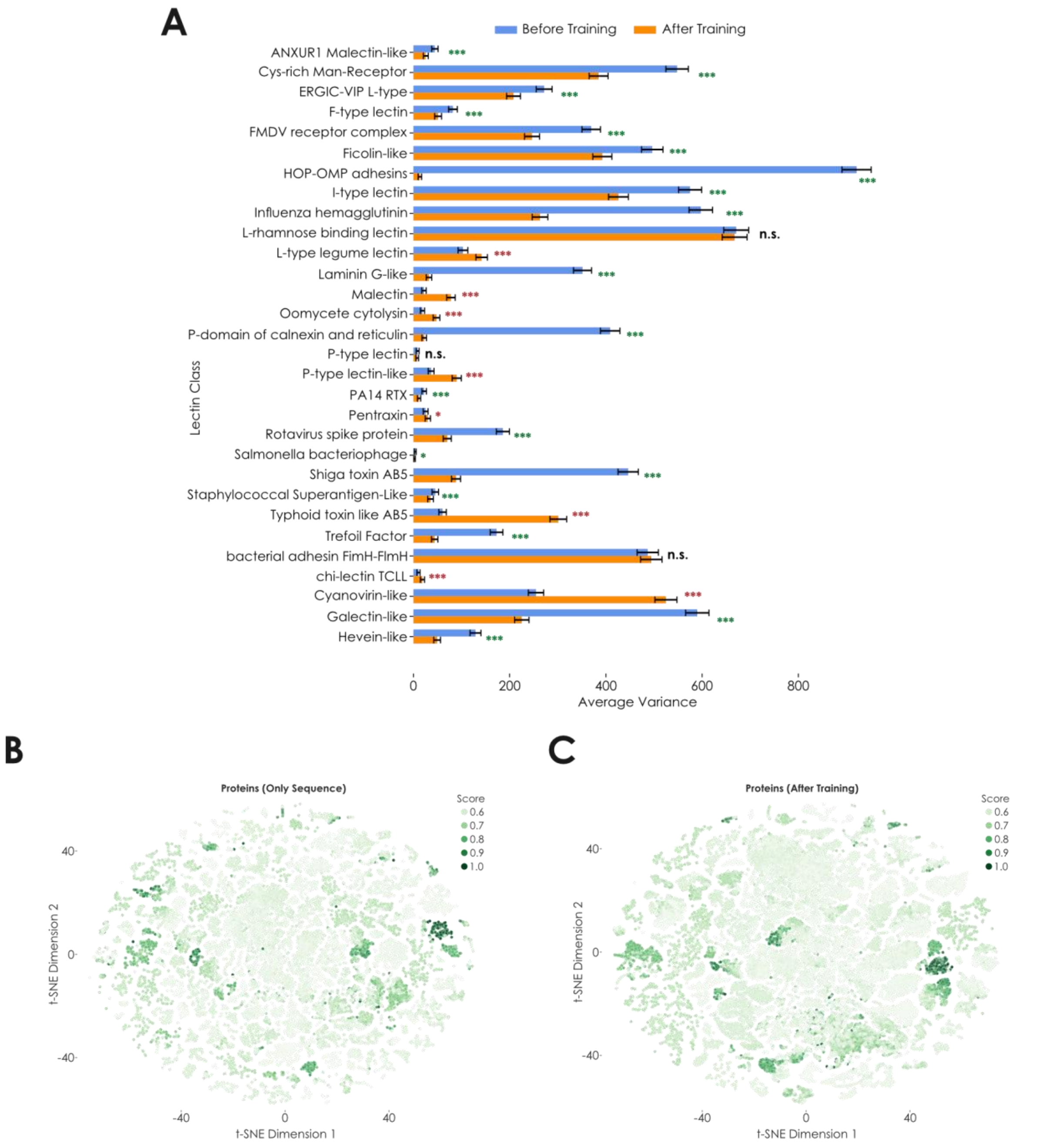

Figure S12. Analyzing putative lectins in LectomeXplore. A) Training LectinOracle improves clustering by lectin class. Variance by lectin class is shown before and after training, with significant differences being tested via an F-test. * $p < 0.05$, ** $p < 0.01$, *** $p < 0.001$. **B-C)** Lectins from LectomeXplore clustered based on sequence similarity or binding specificity. Learned representations from the pre-trained ESM-1b model (B) or a trained LectinOracle model (C) were extracted for all 120,523 putative lectins in LectomeXplore with a similarity score higher than 0.5. Lectins were colored by the sequence similarity score from LectomeXplore.

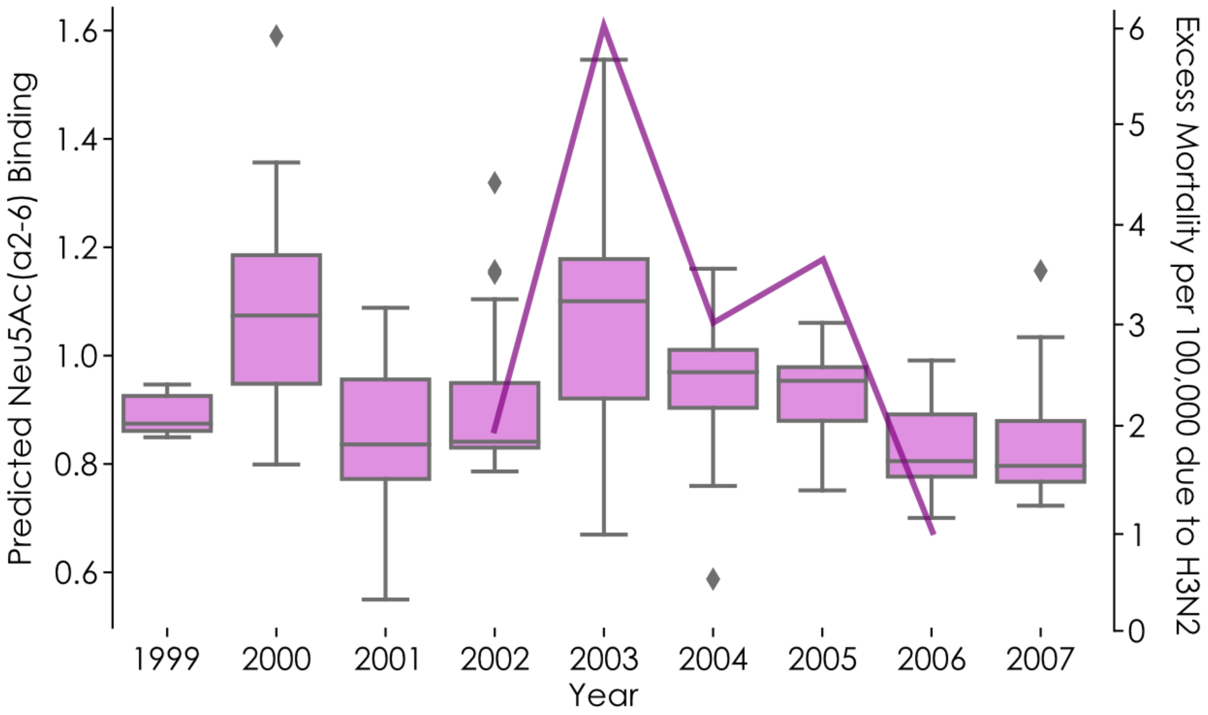

Figure S13. Predicting H3N2 influenza excess mortality with LectinOracle. For each year, from 1999 to 2007, we used the hemagglutinin sequences from yearly strains of H3N2 influenza viruses in Taiwan to generate binding predictions to Neu5Ac(α2-6)-containing glycans with LectinOracle, shown as a boxplot, depicting mean, quartiles, and data distribution. Then, we overlayed excess mortality due to H3N2 influenza virus in Taiwan from 2002 to 2006 as a lineplot to facilitate comparisons to our predictions.

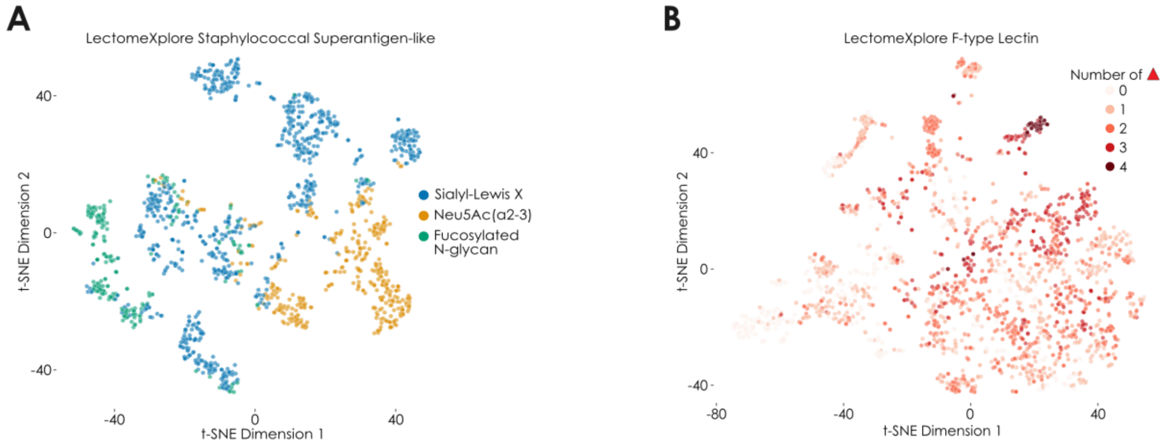

Figure S14. Analyzing LectomeXplore lectin classes with LectinOracle. A-B) We used LectinOracle to predict the binding specificity for all 1,411 staphylococcal superantigen-like (SSL, A) or 2,206 F-type (B) lectins with a score above 0.5 in LectomeXplore. Shown are the learned lectin representations learned by LectinOracle, analogous to Figure 4C, colored in by preferred binding motif. Lectins colored in for “Neu5Ac(α2-3)” binding did not show preferred binding to Sialyl-LewisX motifs. For (B), we counted the number of fucoses in the top five enriched motifs as a measure of fucose binding promiscuity
